# Systemic inflammation impairs myelopoiesis and interferon type I responses in humans

**DOI:** 10.1038/s41590-025-02136-4

**Published:** 2025-04-18

**Authors:** Farid Keramati, Guus P. Leijte, Niklas Bruse, Inge Grondman, Ehsan Habibi, Cristian Ruiz-Moreno, Wout Megchelenbrink, Annemieke M. Peters van Ton, Hidde Heesakkers, Manita E. Bremmers, Erinke van Grinsven, Kiki Tesselaar, Selma van Staveren, Walter J. van der Velden, Frank W. Preijers, Brigit te Pas, Raoul van de Loop, Jelle Gerretsen, Mihai G. Netea, Hendrik G. Stunnenberg, Peter Pickkers, Matthijs Kox

**Affiliations:** 1 Department of Molecular Biology, Faculty of Science, Radboud University, Nijmegen, The Netherlands; 2 Princess Maxima Center for Pediatric Oncology, Utrecht, The Netherlands; 3 Center for Translational Immunology, University Medical Center Utrecht, Utrecht, The Netherlands; 4 Department of Intensive Care Medicine, Radboud University Medical Center, Nijmegen, The Netherlands; 5 Department of Internal Medicine, Radboud University Medical Center, Nijmegen, The Netherlands; 6 Broad Institute of MIT and Harvard, Cambridge, MA USA; 7 Sprott Centre for Stem Cell Research, Regenerative Medicine Program, Ottawa Hospital Research Institute, Ottawa, Québec Canada; 8 Department of Cellular and Molecular Medicine, Faculty of Medicine, University of Ottawa, Ottawa, Québec Canada; 9 Department of Precision Medicine, University of Campania Luigi Vanvitelli, Naples, Italy; 10 Department of Hematology, Radboud University Medical Center, Nijmegen, The Netherlands; 11 Department of Respiratory Medicine and Center of Translational Immunology, University Medical Center Utrecht, Utrecht, The Netherlands; 12 TmonoCOAST, Amsterdam, The Netherlands; 13 Human Genomics Laboratory, Craiova University of Medicine and Pharmacy, Craiova, Romania

**Keywords:** Toll-like receptors, Infectious diseases, Haematopoiesis

## Abstract

Systemic inflammatory conditions are classically characterized by an acute hyperinflammatory phase, followed by a late immunosuppressive phase that elevates the susceptibility to secondary infections. Comprehensive mechanistic understanding of these phases is largely lacking. To address this gap, we leveraged a controlled, human in vivo model of lipopolysaccharide (LPS)-induced systemic inflammation encompassing both phases. Single-cell RNA sequencing during the acute hyperinflammatory phase identified an inflammatory *CD163*^+^*SLC39A8*^+^*CALR*^+^ monocyte-like subset (infMono) at 4 h post-LPS administration. The late immunosuppressive phase was characterized by diminished expression of type I interferon (IFN)-responsive genes in monocytes, impaired myelopoiesis and a pronounced attenuation of the immune response on a secondary LPS challenge 1 week after the first. The infMono gene program and impaired myelopoiesis were also detected in patient cohorts with bacterial sepsis and coronavirus disease. IFNβ treatment restored type-I IFN responses and proinflammatory cytokine production and induced monocyte maturation, suggesting a potential treatment option for immunosuppression.

## Abstract (web summary)

Stunnenberg et al. use a model of lipopolysaccharide injection in humans to characterize the transcriptomic landscape of bone marrow and blood immune cells during the hyperinflammatory and immunosuppressed phases of systemic inflammation.

## Main

Systemic inflammation plays a pivotal role in the pathophysiology of several diseases, such as sepsis and viral infections, including COVID-19, and contributes to 20% of global mortality^1–4^. Systemic inflammation is classically characterized by an acute hyperinflammatory phase, often termed as ‘cytokine storm’, followed by a late immunosuppressive phase^5^ that renders patients susceptible to secondary infections^6^. This sustained refractory state of the immune system is associated with high late-onset mortality^7–10^. Despite the substantial burden on healthcare systems, unraveling the molecular mechanisms underpinning systemic inflammation-related pathogenicity is extremely difficult, as a result of extensive heterogeneity in terms of inflammation onset time, etiology, site of infection and underlying comorbidities. Mouse models of systemic inflammation are ubiquitously used and valuable, but suffer from important interspecies differences and poorly replicate the human inflammatory response^11^, thereby limiting translatability^12^.

The human in vivo model of lipopolysaccharide (LPS)-induced systemic inflammation^13^, also known as experimental endotoxemia, is a tightly controlled systemic inflammation model in which healthy volunteers are injected with bacterial LPS intravenously to elicit transient but profound acute hyperinflammation, followed by a late immunosuppressive phase^14^. Previous studies utilizing the experimental endotoxemia model primarily conducted transcriptomic analyses at the whole-blood level and focused on the hyperinflammatory phase, thereby limiting the ability to dissect unbiased cell-type-specific effects of systemic inflammation and examine the mechanistic underpinnings of the immunosuppressive phase^15^.

In the present study, we combined massively parallel single-cell RNA sequencing (scRNA-seq) and cellular functional assays to characterize the hyperinflammatory and immunosuppressive phases after LPS administration in peripheral blood and bone marrow leukocytes. Leveraging of publicly available large cohorts of patients with sepsis and COVID-19 indicated the clinical relevance of our comprehensive longitudinal dataset. Our results identified conserved gene expression programs, mainly in monocytes and T cells, during the acute LPS-induced hyperinflammatory phase, early sepsis and COVID-19. In the immunosuppressive phase, we observed a significant impairment of monocyte functionality and maturation accompanied by decreased interferon type I (IFN-I) signaling that could be restored by IFNβ treatment.

## Results

### LPS induces hyperinflammation followed by immunosuppression

**Fig. 1:**
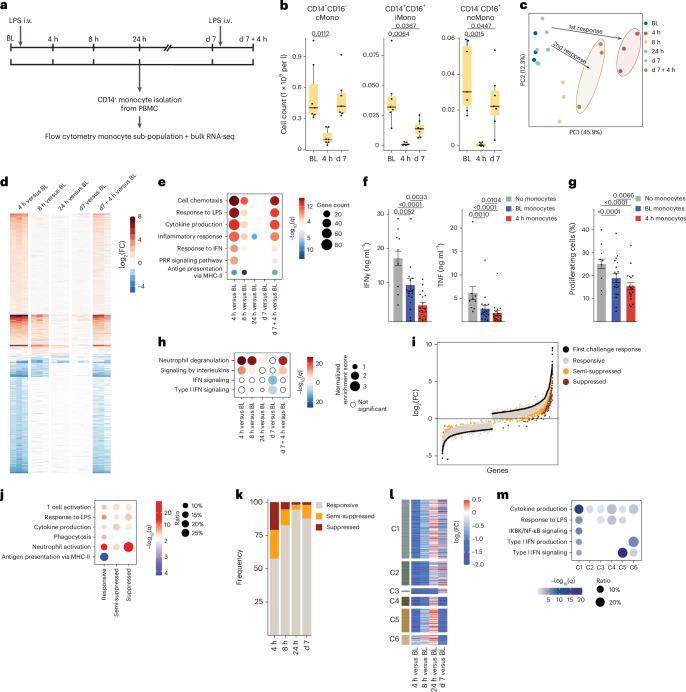
Impairment of IFN-I pathway in blood monocytes 1 week after LPS-induced systemic inflammation. **a**, Schematic representation of the study and sample acquisition in healthy volunteers (*n* = 11). Seven volunteers were intravenously (i.v.) injected with LPS (2 ng kg^−1^) at baseline (BL, d0) and d7. Four volunteers were intravenously injected with placebo (0.9% NaCl) at BL. Peripheral blood was collected at BL, 4 h, 8 h, 24 h, d7 and d7 + 4 h. **b**, Box plots of absolute abundance of CD14^+^CD16^−^ cMonos, CD14^+^CD16^+^ iMonos and CD14^−^CD16^+^ ncMonos in blood of LPS-challenged volunteers (*n* = 7) at BL, 4 h and d7. **c**, Principal component analysis of blood CD14^+^ monocyte transcriptomes from LPS-challenged volunteers (*n* = 3) at BL, 4 h, 8 h, 24 h, d7 and d7 + 4 h. **d**, Heatmap representation of DEGs at 4 h, 8 h, 24 h, d7 and d7 + 4 h compared with BL as in **c**. **e**, GO term analysis of DEGs at 4 h, 8 h, 24 h, d7 and d7 + 4 h compared with BL as in **c**. **f**, Bar plots of TNF and IFNγ production in CD3^+^ T cells isolated from the blood of healthy donors (*n* = 9), activated with CD3 and CD28 antibody-coupled beads and co-cultured with cMonos obtained from LPS-challenged volunteers (*n* = 6) at BL and 4 h at 1:2 ratio. **g**, Bar plots of percentage of proliferating CD3- and CD28-activated CD3^+^ T cells isolated from the blood of healthy donors (*n* = 9), and co-cultured with cMonos obtained from LPS-challenged volunteers (*n* = 6) at BL and 4 h at 1:2 ratio as in **f**. **h**, GSEA of gene expression profiles at 4 h, 8 h, 24 h, d7 and d7 + 4 h compared with BL as in **c**. **i**, DEGs in blood CD14^+^ monocytes from LPS-challenged volunteers (*n* = 3) in response to both LPS challenges (first: 4 h versus BL; second: 7 d + 4 h versus 7 d) clustered into three groups: responsive (FC < 2), semi-suppressed (2 < FC < 3) and suppressed (FC > 3). **j**, GO term analysis of responsive, semi-suppressed and suppressed genes in CD14^+^ monocytes from LPS-challenged volunteers (*n* = 3) as in **j**. **k**, Percentage of responsive (FC < 2), semi-suppressed (2 < FC < 3) and suppressed (FC > 3) DEGs in blood CD14^+^ monocytes from LPS-challenged volunteers (*n* = 3) obtained at 4 h, 8 h, 24 h and d7 that were ex vivo stimulated with LPS (10 ng ml^−1^) versus CD14^+^ LPS-stimulated monocytes obtained at BL. **l**, Heatmap representation of average expression of DEGs in CD14^+^ restimulated monocytes from LPS-challenged volunteers (*n* = 3) obtained at 4 h, 8 h, 24 h and d7 based on fold-change relative to BL (*n* = 3) as in **k**. Genes were clustered (C1–C6) based on their behavior across the different time points. **m**, GO term analysis of genes in clusters C1–C6 defined as in **l**. The box plots in **b** show the median, first and third quartiles and the whiskers 1.5× the interquartile range (IQR). The bar plots in **f** and **g** are presented as mean values ± s.e.m. The *P* values were calculated using two-sided, paired Wilcoxon’s signed-rank tests.

**Extended Data Fig. 1:**
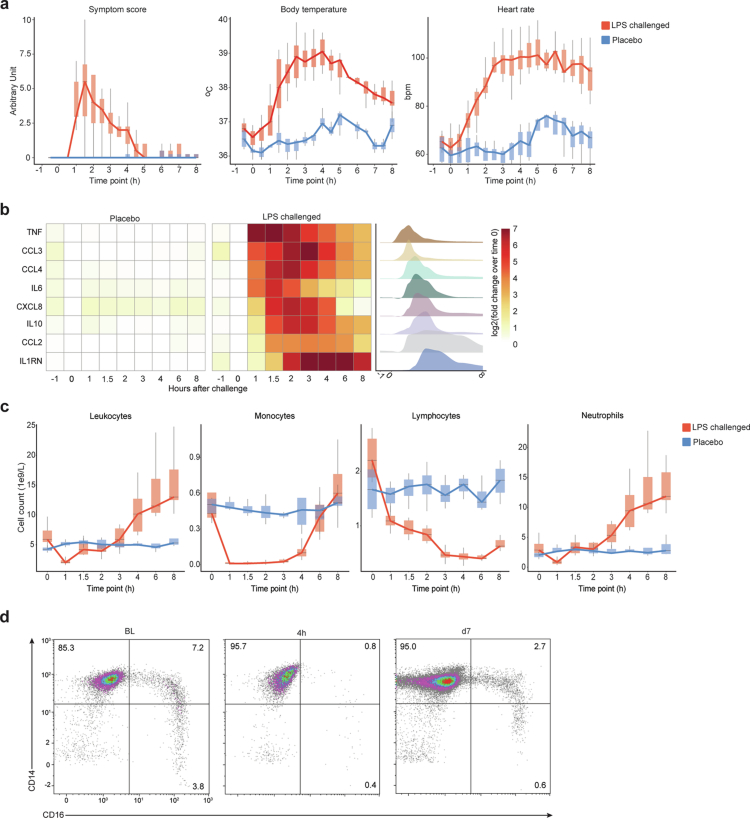
Characterization of LPS-induced systemic inflammation. **a**, Box plots of flu-like symptoms, body temperature, and heart rate following intravenous injection of placebo (0.9% NaCl, blue, n = 4) or LPS (2 ng kg^−1^, red, n = 7) in healthy volunteers. **b**, Heatmap of log2(fold-change) blood concentrations of cytokines TNF, IL-10, CCL4, CCL3, IL-6, CXCL8, CCL2, IL-1RN following intravenous injection of placebo (0.9% NaCl, n = 4) or LPS (2 ng kg^−1^, n = 7) compared with baseline (timepoint 0) in healthy volunteers. **c**, Box plots of absolute number of blood leukocytes, monocytes, lymphocytes and neutrophils following intravenous injection of placebo (0.9% NaCl, blue, n = 4) or LPS (2 ng kg^−1^, red, n = 7) in healthy volunteers. **d**, Representative example of flow cytometric analysis of different blood monocyte subsets before (baseline) and at 4 h and d7 following LPS administration (2 ng kg^−1^). Box plots in **a**, **c** shows median, first and third quartiles; whiskers show 1.5x the interquartile range.

To study systemic inflammation in humans in vivo, healthy male volunteers (Supplementary Table 1) were intravenously injected with 2 ng kg^−1^ of LPS (*n* = 7, age 24 (19–30) years) or placebo (*n* = 4, age 19 (18–28) years; Fig. 1a). The appearance of clinical systemic inflammation symptoms, such as fever and tachycardia (Extended Data Fig. 1a), and a transient profound increase in circulating cytokines, including tumor necrosis factor (TNF), interleukin (IL)-10, CCL4, CCL3, IL-6, CXCL8, CCL2, *IL-1RN* (IL-1ra gene) (Extended Data Fig. 1b) up to 8 h after LPS administration, confirmed the induction of hyperinflammation. Severe monocytopenia was observed at 1 h after LPS administration (Extended Data Fig. 1c), with CD14^+^CD16^−^ classic monocytes (cMonos) starting to repopulate the blood ~3 h post-LPS and returning to baseline levels (defined as immediately before the LPS challenge) at ~6 h post-LPS^16^ (Extended Data Fig. 1c). Between 6 h and day 7 (d7) after the LPS challenge, cMonos gradually differentiated into CD14^+^CD16^+^ intermediate monocytes (iMonos) and CD14^−^CD16^+^ nonclassic monocytes (ncMonos) (Extended Data Fig. 1d)^16^; however, the abundance of these subsets in blood remained significantly decreased at d7 after the LPS challenge (Fig. 1b), suggesting impaired monocyte maturation.

**Extended Data Fig. 2:**
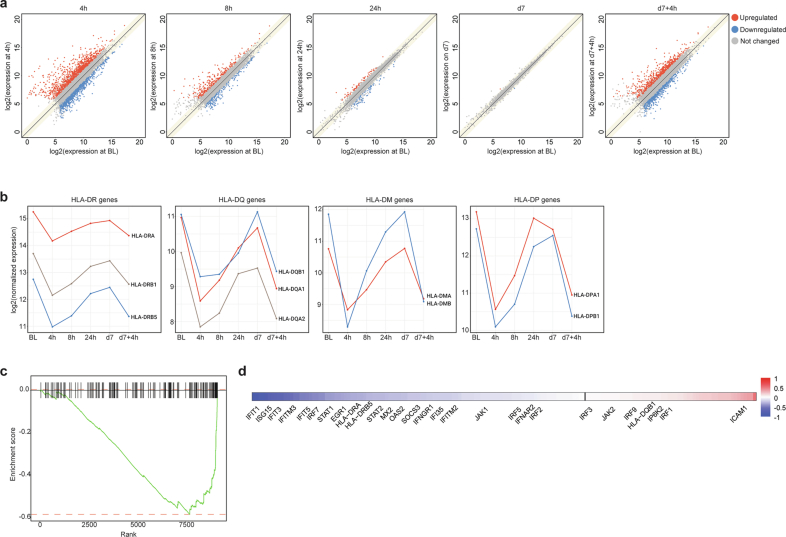
IFN signaling impairment in blood monocytes in the late immunosuppressive phase following LPS administration. **a**, Scatterplot of gene expression in blood CD14^+^ monocytes at 4 h, 8 h, 24 h, d7, and d7 + 4 h following LPS administration (y-axis) versus baseline (before LPS administration, x-axis) in healthy volunteers. Dots represent the average expression of n = 3 replicates. Red dots indicate significantly up-regulated genes (fold-change > 2 & q value < 0.05), while blue dots depict significantly down-regulated genes (fold-change < 0.5 & q value < 0.05). **b**, log2(normalized expression) of MHC class II (HLA-II) genes in blood CD14^+^ monocytes at baseline (BL, before LPS administration) and at 4 h, 8 h, 24 h, d7, and d7 + 4 h following LPS administration in healthy volunteers (n = 3). **c**, Gene set enrichment analysis of interferon signaling genes in blood CD14^+^ monocytes of LPS-challenged healthy volunteers (n = 3, based on the d7 vs. BL comparison). **d**, log2(relative normalized expression) of interferon signaling genes in blood CD14^+^ monocytes of LPS-challenged healthy volunteers (n = 3, ordered and colored based on relative expression of d7 vs. BL).

Bulk RNA-seq of blood CD14^+^ monocytes obtained from LPS-challenged volunteers (*n* = 3) showed significant perturbation at 4 h and 8 h post-LPS compared with baseline (1,459 upregulated and 1,222 downregulated genes), which substantially normalized at 24 h post-LPS (Fig. 1c), with no significant differentially expressed genes (DEGs) at d7 compared with baseline (Fig. 1d and Extended Data Fig. 2a). Gene ontology (GO) of upregulated DEGs at 4–8 h post-LPS was mainly attributed to inflammatory response (*CXCL8* and *IL-6*) and IFN type I (IFN-I) signaling pathways (*MX1* and *IRF7*) (Fig. 1e), whereas antigen presentation through major histocompatibility complex (MHC) class II was the most significant GO term of downregulated genes at 4 h (*HLA-DRA* and *HLA-DRB5*) (Fig. 1e and Extended Data Fig. 2b). As decreased expression of the MHC-I receptor human leukocyte antigen isotype DR (HLA-DR) in CD14^+^ monocytes indicates the induction of T cell inhibitory, myeloid-derived suppressor cells (MDSCs), a correlate of impaired outcomes in patients with sepsis^17,18^, we co-incubated blood cMonos obtained from LPS-challenged volunteers at baseline and at 4 h post-LPS (*n* = 6) with naive blood CD3^+^ T cells obtained from non-LPS-challenged healthy donors (*n* = 9) in the presence of CD3 and CD28 antibody-coupled beads to induce cytokine production and proliferation through T cell receptor stimulation. Co-incubation with cMonos obtained 4 h post-LPS strongly inhibited IFNγ and TNF secretion (Fig. 1f) and decreased T cell proliferation (Fig. 1g) compared with co-incubation with cMonos obtained at baseline, suggesting that they were immunosuppressive. Gene set enrichment analysis (GSEA) indicated a significant downregulation of IFN-I signaling at d7 compared with baseline in CD14^+^ monocytes (Fig. 1h). Nevertheless, very few genes were differentially expressed between these time points and, although the expression of several IFN-stimulated genes (ISGs) was lower on d7 compared with baseline, this did not reach statistical significance at the level of individual genes (Extended Data Fig. 2c,d and Supplementary Table 2).

**Extended Data Fig. 3:**
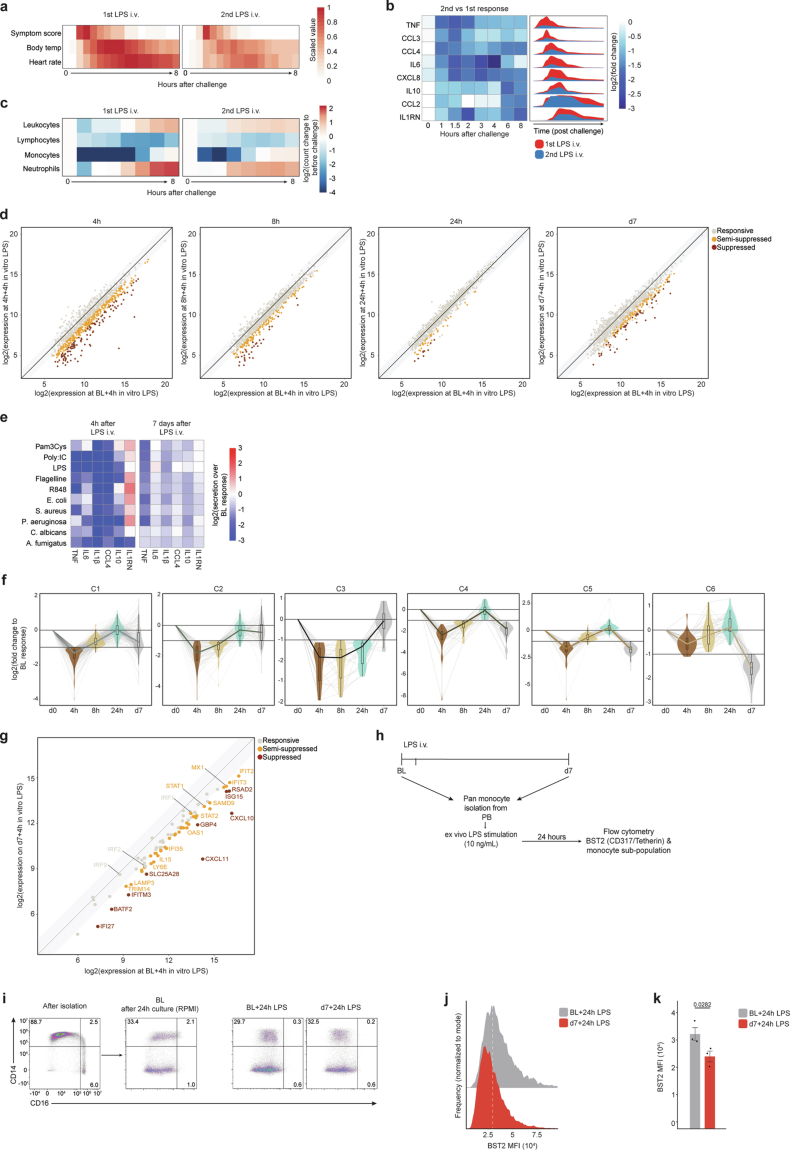
Immunosuppression in the late immunosuppressive phase following LPS administration. **a**, Heatmap of changes in flu-like symptoms, body temperature, and heart rate after the first and second intravenous challenge with 2 ng kg^−1^ LPS in healthy volunteers (n = 7). **b**, Heatmap of differences in log2(fold-change) blood concentrations of cytokines TNF, IL-10, CCL4, CCL3, IL-6, CXCL8, CCL2, IL-1RN between the first and second intravenous challenge with 2 ng kg^−1^ LPS in healthy volunteers (n = 7). **c**, Heatmap of changes in blood leukocyte (subtypes) after the first and second intravenous challenge with 2 ng kg^−1^ LPS in healthy volunteers (n = 7). **d**, Scatterplot of mean normalized gene expression profiles of blood CD14^+^ monocytes from LPS-challenged volunteers (n = 3) obtained at 4 h, 8 h, 24 h, and d7 that were *ex vivo* stimulated with LPS (10 ng mL^−1^) vs. CD14^+^ LPS-stimulated monocytes obtained at BL. Orange dots indicate semi-suppressed genes (-1>log2[fold-change]>-2) and red dots indicate suppressed genes (-2>log2[fold-change]). **e**, Heatmap of average log2(fold-change) difference in cytokine (TNF, IL6, IL1β, CCL4, IL10, IL1RN) production by blood CD14^+^CD16^−^ classical monocytes (cMonos) obtained at 4 h and d7 after LPS administration in healthy volunteers (n = 7) that were *ex vivo* stimulated with Pam3Cys, Poly:IC, LPS, Flagelline, R848, *E. coli*, *S. aureus*, *P. aeruginosa*, *C. albicans*, and *A. fumigatus* compared with cytokine production of cMonos obtained from the same subjects at baseline (before LPS administration) stimulated with the same ligands. **f**, violin plot of log2(fold-change) difference in gene expression of blood CD14^+^ monocytes from LPS-challenged volunteers (n = 3) obtained at 4 h, 8 h, 24 h, and d7 that were *ex vivo* stimulated with LPS (10 ng mL^−1^) vs. CD14^+^ LPS-stimulated monocytes obtained at BL. Genes are clustered based on their dynamics over time (see main text and Fig. 1l). **g**, Scatterplot of log2(normalized expression) of IFN-I signaling pathway genes in blood CD14^+^ monocytes from LPS-challenged volunteers obtained at d7 that were *ex vivo* stimulated with LPS (10 ng mL^−1^, y-axis, mean expression of n = 3) compared to LPS-stimulated CD14+ monocytes form the same volunteers obtained at baseline (x-axis, mean expression of n = 3). **h**, Schematic representation of *ex vivo* BST2 flow cytometry experiments in immunosuppressed pan monocytes (containing all subsets) **i**, Representative example of flow cytometric analysis of monocyte subsets immediately after isolation, and after 24 h of incubation with or without LPS (10 ng mL^−1^). **j**, Overlay of BST2 mean fluorescence intensity (MFI) in cMonos obtained at baseline and at d7 after 24 h of *ex vivo* stimulation with LPS (10 ng mL^−1^). **k**, Bar plot of BST2 MFI in cMonos (n = 3) obtained at baseline and at d7 after 24 h of *ex vivo* stimulation with LPS (10 ng mL^−1^). Bar plot in **k** presented as mean values +/− SEM. The *P* value calculated using a paired two-sided t-test.

To functionally investigate the late immunosuppressive phase, LPS-challenged volunteers (*n* = 7) were intravenously re-challenged with the same dose of LPS at d7. During the 8 h after this second LPS challenge, we observed markedly less pronounced clinical symptoms and TNF, IL-10, CCL4, CCL3, IL-6, CXCL8, CCL2 and IL-1RN elevation compared with the same timeframe after the first challenge (Extended Data Fig. 3a–c). Bulk RNA-seq on blood CD14^+^ monocytes obtained from LPS-challenged volunteers (*n* = 3) indicated that almost all DEGs (93.6%) from 4 h post-first LPS challenge showed a less pronounced response at 4 h post-second LPS challenge, with an average 30% decrease in responsiveness (Fig. 1c,d and Supplementary Table 3). Based on the difference in gene expression between CD14^+^ monocytes obtained 4 h after the first and second challenges, DEGs clustered into three groups: responsive (fold-change (FC) < 2, *EREG* and *ACSL1*), semi-suppressed (2 < FC < 3, *OAS3* and *IFITM2*) and suppressed (FC > 3, *CCL4* and *NFKB2*), with 15% of DEGs falling into the suppressed and semi-suppressed clusters (Fig. 1i). GO terms associated with chemokine-mediated response and neutrophil activation were suppressed, whereas phagocytosis remained functional 4 h after the second challenge compared with 4 h after the first challenge (Fig. 1j).

Next, bulk RNA-seq on blood CD14^+^ monocytes obtained from LPS-challenged volunteers (*n* = 3) at baseline and at 4, 8, 24 h and d7 post-LPS that were restimulated ex vivo with LPS for 4 h indicated that DEGs could be classified into responsive (FC < 2), semi-suppressed (2 < FC < 3) and suppressed (FC > 3). Approximately 43% of DEGs at 4 h and 17% of DEGs at 8 h were semi-suppressed or suppressed (Fig. 1k). The response of CD14^+^ monocytes at 24 h was similar to that at baseline (6% (semi-)suppressed DEGs), whereas 12% of DEGs on d7 were semi-suppressed or suppressed compared with baseline (Fig. 1k and Extended Data Fig. 3d). Stimulation of blood cMonos obtained from LPS-challenged volunteers (*n* = 7) at baseline and at 4 h and d7 post-LPS administration with various pathogen-associated molecular patterns Pam3Cys, poly(inosinic:cytidylic acid) (poly(I:C)), LPS, flagellin and R848, and heat-killed pathogens *Escherichia coli, Staphylococcus aureus, Pseudomonas aeruginosa* and *Candida albicans* for 24 h indicated a marked attenuation in the production of TNF, IL-6, IL-1β, CCL4, IL-10 and IL-1RN at 4 h post-LPS and a less pronounced attenuation at d7 post-LPS compared with baseline (Extended Data Fig. 3e), suggesting broad immunosuppression in response to various triggers, not just LPS. Based on their dynamics at 4 h, 8 h, 24 h and d7 post-LPS administration, DEGs in LPS-restimulated CD14^+^ monocytes were classified into six clusters (C1–C6; Fig. 1l and Extended Data Fig. 3f). GO analysis indicated that the cytokine production pathway was suppressed in all clusters (Fig. 1n). GO terms and individual genes related to the IFN-I signaling pathway were suppressed in LPS-restimulated CD14^+^ monocytes obtained at d7 (clusters C5 and C6) (Fig. 1l and Extended Data Fig. 3g). Ex vivo stimulation of blood pan-monocytes (containing all subsets) obtained from LPS-challenged volunteers at baseline and at d7 with LPS for 24 h (*n* = 3) resulted in loss of iMonos and ncMonos, in contrast to samples cultured without LPS, which retained iMonos (Extended Data Fig. 3h,i). Blood cMonos obtained at d7 post-LPS administration that were ex vivo stimulated with LPS exhibited significantly reduced expression of the IFN-induced protein BST2 compared with LPS-stimulated cMonos obtained at baseline (Extended Data Fig. 3j,k).

Collectively, these results indicated an initial hyperinflammatory phase up to 8 h after an LPS challenge, followed by an immunosuppressive phenotype apparent at d7 post-LPS. The substantially attenuated in vivo and ex vivo monocyte responses at d7 suggested immunological rewiring in monocytes.

### LPS triggers inflammatory gene programs in myeloid and lymphoid cells

**Fig. 2:**
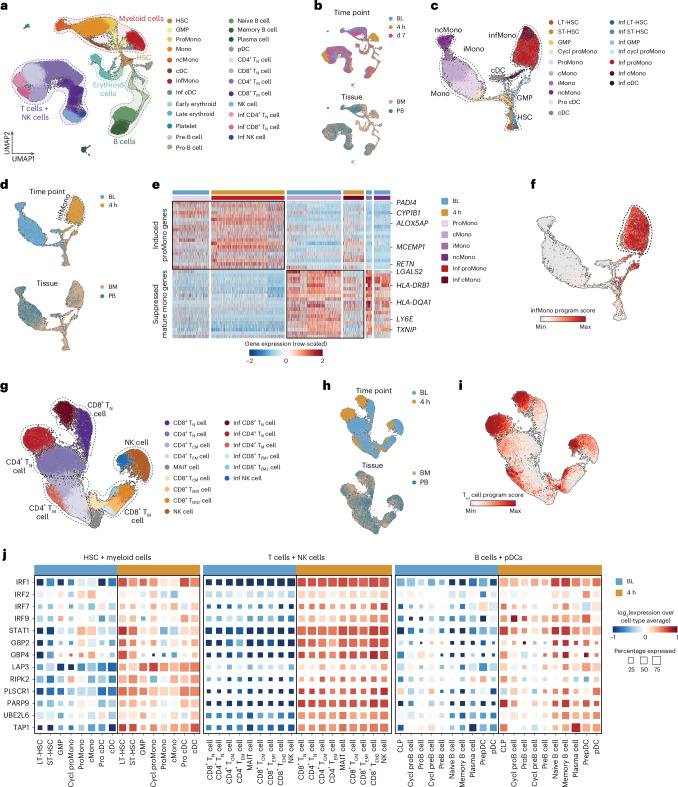
Single-cell characterization of the acute hyperinflammatory response to LPS administration. **a**, UMAP representation of all single cells obtained from bone marrow and peripheral blood of LPS-challenged healthy volunteers (*n* = 3 for each compartment at BL: bone marrow, 7 d before the LPS challenge; blood, immediately before the LPS challenge) and at 4 h and d7 post-LPS challenge, colored based on cell type. **b**, UMAP of all blood and bone marrow cells (*n* = 3) as in **a**, colored based on acquisition time point (top) and collected compartment (bottom). **c**, UMAP of blood and bone marrow HSCs and myeloid lineage cells at BL and 4 h (*n* = 3) as in **a**, colored based on cell type. **d**, UMAP of blood (PB) and bone marrow (BM) HSCs and myeloid lineage cells at BL and 4 h (*n* = 3) as in **c**, colored based on time point (top) and compartment (bottom). **e**, Heatmap representation of pro-monocyte and mature monocyte genes in myeloid cells from the bone marrow and blood of LPS-challenged healthy volunteers (*n* = 3) at BL and 4 h. **f**, NMF-inferred gene program enriched in blood and bone marrow infMonos from bone marrow and blood of LPS-challenged healthy volunteers (*n* = 3) as in **d**. **g**, UMAP of blood and bone marrow T and NK cells at BL and 4 h (*n* = 3) as in **a**, colored based on cell type. **h**, UMAP of blood and bone marrow T and NK cells at BL and 4 h (*n* = 3) as in **g**, colored based on time point (top) and compartment (bottom). **i**, NMF-inferred gene program enriched in T_inf_ cells from bone marrow and blood of LPS-challenged healthy volunteers (*n* = 3) as in **h**. **j**, Normalized expression profile of several IFN pathway genes in blood and bone marrow HSCs and myeloid lineage cells, T cells and NK cells, and B cells and pDCs of LPS-challenged healthy volunteers at BL and 4 h (*n* = 3). Max, maximum; min, minimum; Cycl, cycling; T_N_, naive T cell; T_CM_, central memory T cell; T_EM_, effector memory T cell; MAIT, mucosal-associated invariant T cell.

**Extended Data Fig. 4:**
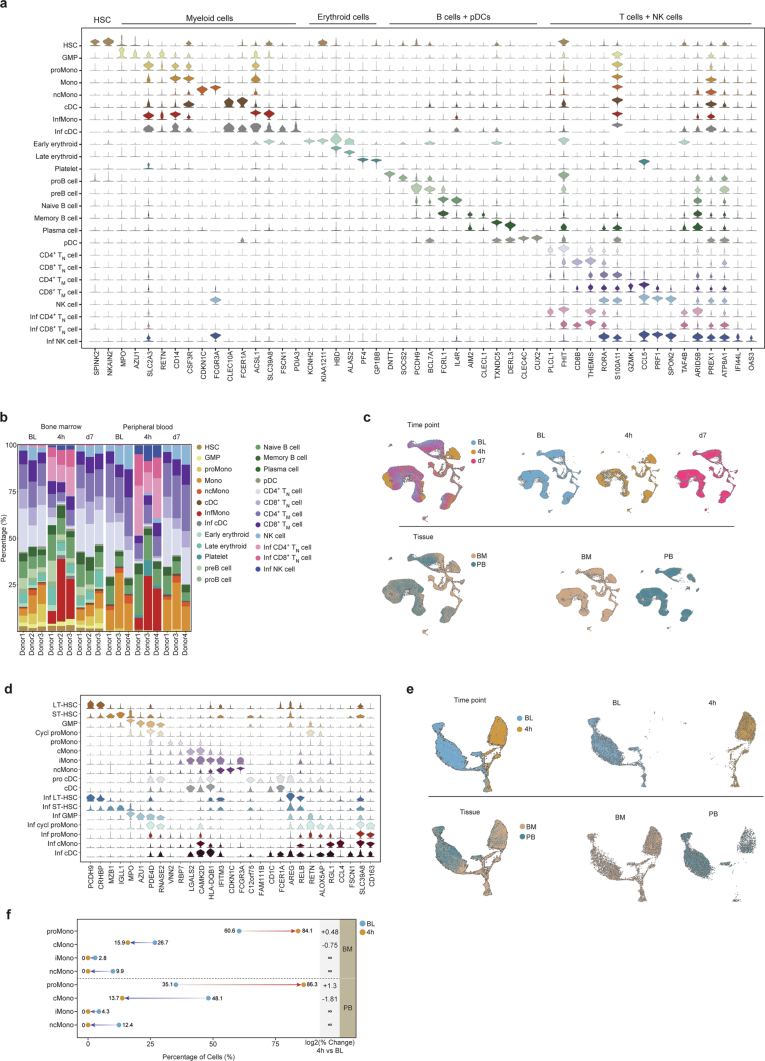
Single-cell characterization of the acute hyperinflammatory response to LPS administration in peripheral blood and bone marrow cells. **a**, Violin plot of marker genes of all mononuclear immune cell types identified in peripheral blood and bone marrow obtained from LPS-challenged healthy volunteers (n = 3 for each compartment) at baseline (BL, before the LPS challenge) and at 4 h and d7 post-LPS challenge. **b**, Sample composition (in percentage) for each cell type depicted for each donor and timepoint as in a. **c**, Overlayed and separated UMAP of all cells in blood and bone marrow obtained from LPS-challenged healthy volunteers (n = 3 for each compartment) at BL, and at 4 h and d7 post-LPS challenge, colored based on acquisition time point (upper) and compartment (bottom). **d**, Violin plot of marker genes HSCs and myeloid cell types identified in blood and bone marrow obtained from LPS-challenged healthy volunteers (n = 3 for each compartment) at BL and at 4 h and d7 post-LPS challenge. **e**, Overlayed and separated UMAP of HSCs and myeloid cells in blood and bone marrow obtained from LPS-challenged healthy volunteers (n = 3 for each compartment) at BL, and at 4 h post-LPS challenge, colored based on acquisition time point (upper) and compartment (bottom). **f**, Percentage of each monocytic cell type in blood and bone marrow obtained from LPS-challenged healthy volunteers (n = 3 for each compartment) at BL and 4 h post-LPS, separated based on compartment.

To determine the acute hyperinflammatory and late immunosuppressive phase changes in the transcriptomic profiles of bone marrow-residing mononuclear cells in LPS-challenged volunteers, bone marrow aspirated from the posterior iliac crest and peripheral blood samples were obtained at baseline (bone marrow, 7 d before the LPS challenge; blood, immediately before the LPS challenge) and at 4 h and d7 post-LPS challenge (*n* = 3), and scRNA-seq was performed using the 10x Genomics platform. After quality control and in silico removal of potential doublets, we obtained the transcriptomic profile of 116,183 cells across the 3 time points and 2 compartments (Supplementary Table 4). Uniform manifold approximation and projection (UMAP)^19^ embedding identified mononuclear cells (Fig. 2a and Extended Data Fig. 4a), with relatively similar proportions of each cell type between donors (Extended Data Fig. 4b). Based on hematopoietic lineage, cells were partitioned into: hematopoietic stem cells (HSCs) and myeloid cells; B cells and plasmocytic dendritic cells (pDCs); T cells and natural killer (NK) cells, and megakaryocyte and erythroid cells; the last of these did not show robust differences in RNA profiles across all time points (Fig. 2b and Extended Data Fig. 4c).

Subclustering and batch correction of the HSCs and myeloid cells cluster revealed a strong displacement of the entire lineage at 4 h compared with baseline, with a prominent emergence of *CD163*^+^*SLC39A*^+^*CALR*^+^ monocyte-like cells (infMonos) at 4 h at the expense of cMonos present as baseline (Fig. 2c,d and Extended Data Fig. 4d,e). Expression of pro-monocyte signature genes, such as *RETN* and *ALOX5AP*, was increased in the infMono cluster, whereas expression of mature monocyte markers such as MHC-II genes was reduced (Fig. 2e). The enrichment of the pro-monocyte signature was accompanied by an increased proportion of *RETN*^+^ pro-monocytes at 4 h (Extended Data Fig. 4f), indicating the emergence of newly generated immature monocytes in the bone marrow and peripheral blood at 4 h post-LPS.

**Extended Data Fig. 5:**
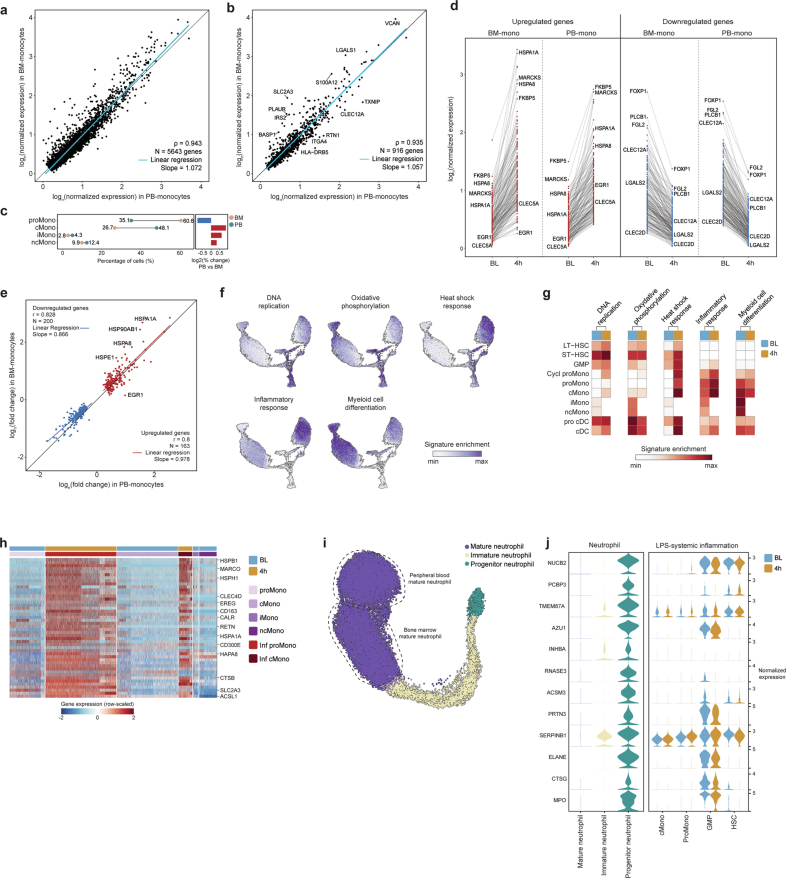
Single-cell characterization of the acute hyperinflammatory response to LPS administration in peripheral blood and bone marrow HSC & myeloid cells. **a**, Scatterplot of normalized overall gene expression profiles of peripheral blood (x-axis) and bone marrow (y-axis) monocytes obtained from healthy volunteers (n = 3) at baseline (before LPS administration). **b**, Scatterplot of normalized baseline monocytic genes (genes highly expressed in monocytes) in blood (x-axis) and bone marrow (y-axis) monocytes obtained from healthy volunteers (n = 3) at baseline (before LPS administration). **c**, Percentage of each monocytic cell type in blood and bone marrow obtained from healthy volunteers (n = 3) at baseline (before LPS administration), colored based on compartment. **d**, Normalized expression of differentially expressed genes in blood and bone marrow monocytes obtained from healthy volunteers (n = 3) at 4 h post-LPS vs. baseline (BL). **e**, Scatterplot of log_n_(fold change) in differentially expressed genes in blood (x-axis) and bone marrow (y-axis) monocytes obtained from healthy volunteers (n = 3) at 4 h post-LPS vs. baseline. **f**, UMAP representation of identified 5 gene sets in blood and bone marrow HSC & myeloid cells of LPS-challenged healthy volunteers (n = 3 for each compartment) at baseline and at 4 h post-LPS challenge, with relative enrichment on single cell level. **g**, Heatmap of average enrichment of 5 identified gene sets in blood and bone marrow HSC & myeloid cells of LPS-challenged healthy volunteers (n = 3 for each compartment) at baseline and at 4 h post-LPS challenge per cell type and timepoint. **h**. Heatmap representation of infMono genes in myeloid cells of LPS-challenged healthy volunteers (n = 3 for each compartment) at baseline and at 4 h post-LPS challenge. **i**, UMAP representation of neutrophils from a publicly available healthy subjects peripheral blood neutrophil dataset. **j**, Violin plot of gene expression profiles of neutrophil-enriched genes in blood and bone marrow HSC & myeloid cells of LPS-challenged healthy volunteers (n = 3 for each compartment) at baseline and at 4 h post-LPS challenge.

The gene expression profile of bone marrow and blood *LYZ*^+^ monocytes showed a high degree of similarity at baseline (Extended Data Fig. 5a,b), although blood monocytes had a slightly higher expression of mature monocyte genes, such as *HLA-DRB5* and *CLEC12A* (Extended Data Fig. 5b). *RETN*^+^ pro-monocytes were the most abundant subset in the bone marrow at baseline, whereas cMonos, iMonos and ncMonos were more abundant in blood at this time point (Extended Data Fig. 5c). DEGs at 4 h post-LPS compared with baseline were consistent between bone marrow and blood monocytes, with nearly all DEGs overlapping and having relatively similar fold-changes (Extended Data Fig. 5d,e).

Single-sample GSEA (ssGSEA) identified five main gene sets (DNA replication, oxidative phosphorylation, heat shock response, inflammatory response and myeloid cell differentiation) that were enriched throughout the myeloid lineage (Extended Data Fig. 5f), with heat shock response exhibiting the highest enrichment in all HSCs and myeloid cell types at 4 h post-LPS compared with baseline (Extended Data Fig. 5g). An unsupervised non-negative matrix factorization (NMF) defined a gene program mainly comprising inflammatory (*CD14* and *LITAF*), heat shock, IFN response (*HSPA1A* and *HSPB1*) and immature pro-monocyte genes (*RETN*) that were specifically enriched in infMonos (Fig. 2f, Extended Data Fig. 5h and Supplementary Table 5).

In inflamed mice, granulocyte–monocyte progenitor (GMP)-derived monocytes that express elevated levels of neutrophil-associated genes such as *MPO* and *ELANE*^20^ are generated predominantly, at the expense of monocyte–dendritic cell progenitor (MDP)-derived monocytes. Leverage of a public human neutrophil dataset^21^ (Extended Data Fig. 5i) indicated that neutrophil-enriched genes (for example, *MPO* and *AZU1)* were not significantly increased in human *LYZ*^+^ monocytes (Extended Data Fig. 5j) at 4 h post-LPS.

**Extended Data Fig. 6:**
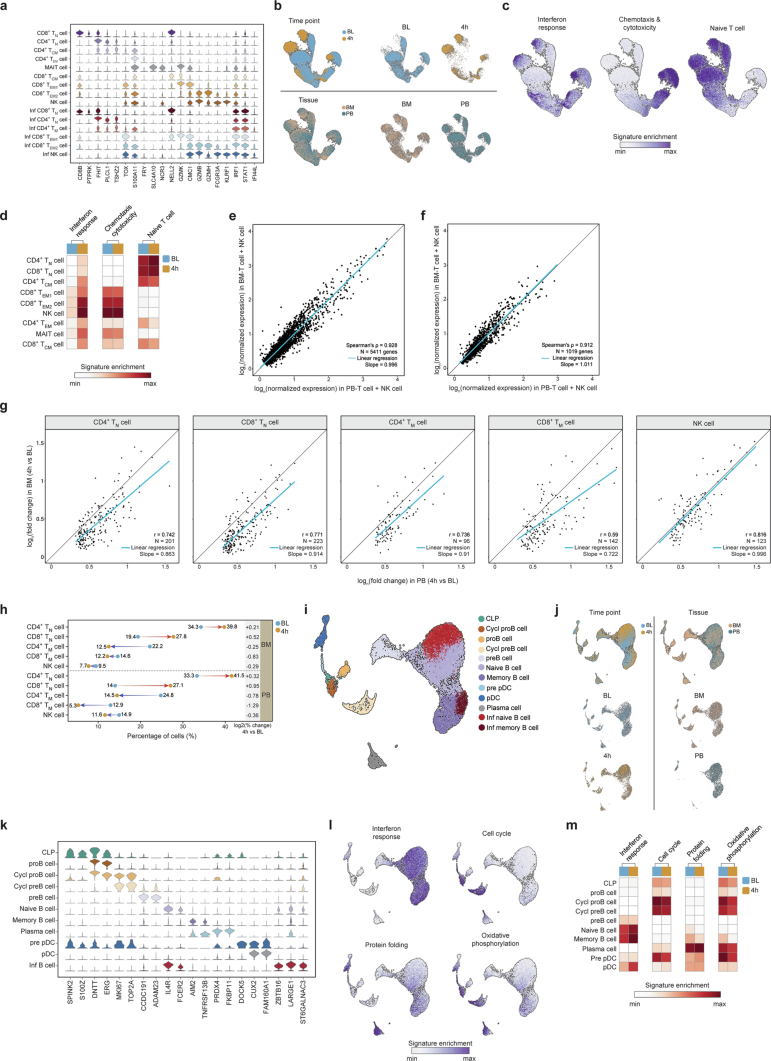
Single-cell characterization of the acute hyperinflammatory response to LPS administration in peripheral blood and bone marrow lymphoid T, B and NK cells. **a**, Violin plot of marker genes of different T and NK cell types identified in samples obtained from LPS-challenged healthy volunteers (n = 3 for each compartment) at baseline (BL, before the LPS challenge) and at 4 h post-LPS challenge. **b**, Overlayed and separated UMAP of lymphoid T and NK cells in blood and bone marrow obtained from LPS-challenged healthy volunteers (n = 3 for each compartment) at BL, and at 4 h post-LPS challenge, colored based on acquisition time point (upper) and compartment (bottom). **c**, UMAP representation of identified 3 gene sets in T and NK cells of LPS-challenged healthy volunteers (n = 3 for each compartment) at baseline and at 4 h post-LPS challenge, with relative enrichment on single cell level. **d**, Heatmap of average enrichment of 3 identified gene sets as in c, per cell type and timepoint. **e**, Scatterplot of normalized overall gene expression profiles of peripheral blood (x-axis) and bone marrow (y-axis) T and NK cells obtained from healthy volunteers (n = 3) at baseline (before LPS administration). **f**, Scatterplot of normalized T and NK cell genes (genes highly expressed in T and NK cells) in blood (x-axis) and bone marrow (y-axis) T and NK cells obtained from healthy volunteers (n = 3) at baseline (before LPS administration). **g**, Scatterplot of log_n_(fold change) in differentially expressed genes in blood (x-axis) and bone marrow (y-axis) T and NK cells obtained from healthy volunteers (n = 3) at 4 h post-LPS vs. baseline. **h**, Percentage of each T and NK cell type in blood and bone marrow samples as in b (n = 3 for each compartment) at BL and 4 h post-LPS, separated based on compartment. **i**, UMAP of B cells and pDCs in blood and bone marrow obtained from LPS-challenged healthy volunteers (n = 3 for each compartment) at BL, and at 4 h post-LPS challenge, colored by cell type. **j**, Overlayed and separated UMAP of lymphoid B and pDC cells in blood and bone marrow obtained from LPS-challenged healthy volunteers (n = 3 for each compartment) at BL, and at 4 h post-LPS challenge, colored based on acquisition time point (left) and compartment (right). **k**, Violin plot of marker genes of different lymphoid B and pDC cells identified in peripheral blood and bone marrow obtained from LPS-challenged healthy volunteers (n = 3 for each compartment) at baseline and at 4 h and d7 post-LPS challenge. **l**, UMAP representation of identified 4 gene sets in blood and bone marrow lymphoid B and pDC cells of LPS-challenged healthy volunteers (n = 3 for each compartment) at baseline and at 4 h post-LPS challenge, with relative enrichment on single cell level. **m**, Heatmap of average enrichment of 4 identified gene sets in blood and bone marrow lymphoid B and pDC cells of LPS-challenged healthy volunteers (n = 3 for each compartment) at baseline and at 4 h post-LPS challenge per cell type and timepoint.

We subclustered, batch corrected and visualized the *CD3*^+^ T cell and *NCAM1*^+^ NK cell subsets (Fig. 2g,h and Extended Data Fig. 6a,b). SsGSEA showed that the IFN-I signaling signature caused the UMAP shift in these subsets at 4 h post-LPS compared with baseline (Extended Data Fig. 6c,d). NMF defined a gene program (inflammatory T cells (T_inf_ cells)) that consisted of IFN-I responsive genes (for example, *MX1*, *IRF1* and *ISG15*) induced in T and NK cells at 4 h (Fig. 2i and Supplementary Table 5). Highly similar gene expression profiles were detected in bone marrow and blood T and NK cells (Extended Data Fig. 6e,f). Naive and memory CD4^+^ and CD8^+^ T cells in the blood exhibited a stronger response to LPS compared with their bone marrow equivalents (Extended Data Fig. 6g), whereas NK cells demonstrated comparable responsiveness in both compartments (Extended Data Fig. 6g). The proportion of naive CD4^+^ and CD8^+^ T cells was substantially increased at 4 h relative to baseline in both compartments, whereas the abundance of memory CD4^+^ and CD8^+^ T cells and NK cells declined (Extended Data Fig. 6h). Analysis of the B cell and pDC clusters showed a slight, yet noticeable, LPS-induced response in B cells at 4 h (Extended Data Fig. 6i–k). SsGSEA revealed four major signatures (IFN signaling, cell cycle, protein folding and oxidative phosphorylation) in B cells at 4 h post-LPS compared with baseline (Extended Data Fig. 6l). The IFN-signaling signature was upregulated in inflammatory B cells and pDCs at 4 h post-LPS compared with baseline (Extended Data Fig. 6m), similar to other lineages (Fig. 2j). Together, these observations indicated that LPS-induced specific signature gene programs were induced in monocytes and T cells during the acute hyperinflammatory phase after LPS administration.

### LPS-induced gene programs are present in sepsis and COVID-19

**Fig. 3:**
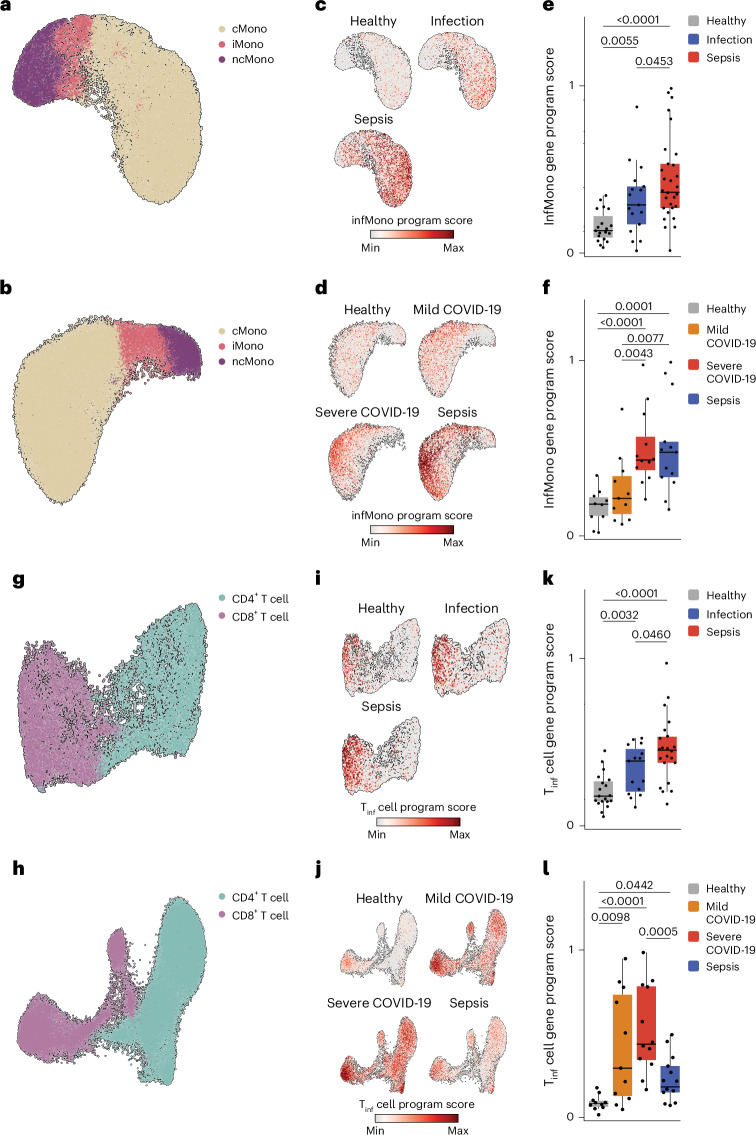
LPS-induced gene programs in sepsis and COVID-19. **a**, UMAP representation of *LYZ*^+^ blood monocytes from patients with early sepsis (*n* = 29) and early nonseptic infection (*n* = 17) and healthy controls (*n* = 19). **b**, UMAP representation of *LYZ*^+^ blood monocytes from patients with early stage mild (*n* = 11) and severe (*n* = 12) COVID-19 and sepsis (*n* = 13) and healthy controls (*n* = 10). **c**, Enrichment of NMF-inferred infMono gene program in *LYZ*^+^ blood monocytes from patients with early sepsis and nonseptic infection and healthy controls as in **a**. **d**, Enrichment of the NMF-inferred infMono gene program in *LYZ*^+^ blood monocytes from patients with early stage mild and severe COVID-19 and sepsis and healthy controls as in **b**. **e**, Box plots of infMono gene program enrichment in *LYZ*^+^ blood monocytes from patients with early sepsis and nonseptic infection and healthy controls as in **a**. **f**, Box plots of infMono gene program enrichment in *LYZ*^+^ blood monocytes from patients with early stage mild and severe COVID-19 and sepsis and healthy controls as in **b**. **g**, UMAP representation of *CD3*^+^ blood T cells from patients with early sepsis and early nonseptic infection and healthy controls as in **a**. **h**, UMAP representation of *CD3*^+^ blood T cells from patients with early stage mild and severe COVID-19 and sepsis and healthy controls as in **b**. **i**, Enrichment of NMF-inferred T_inf_ cell gene program in *CD3*^+^ blood T cells from patients with early sepsis and early nonseptic infection and healthy controls as in **g**. **j**, Enrichment of NMF-inferred T_inf_ cell gene program in *CD3*^+^ blood T cells from patients with early stage mild and severe COVID-19 and sepsis and healthy controls as in **h**. **k**, Box plots of the T_inf_ cell gene program enrichment in *CD3*^+^ blood T cells from patients with early sepsis and nonseptic infection and healthy controls as in **g**. **l**, Box plots of T_inf_ cell gene program enrichment in in *CD3*^+^ blood T cells from patients with early stage mild and severe COVID-19 and sepsis and healthy controls as in **h**. The box plots in **e**, **f**, **k** and **l** show the median, first and third quartiles and the whiskers 1.5× the IQR. The *P* values were calculated using two-sided, unpaired Wilcoxon’s signed-rank tests.

**Extended Data Fig. 7:**
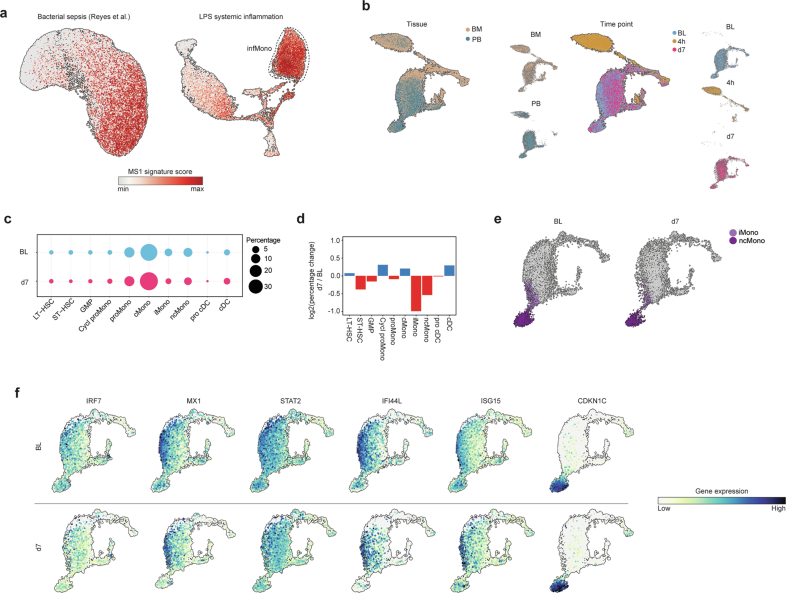
LPS-induced gene programs in patients with early sepsis and impaired monocyte maturation in the late immunosuppressive phase following LPS administration. **a**, UMAP representation of enrichment of sepsis specific MS1 (MDSC) gene signature defined in *LYZ*^*+*^ monocytes of patients with early sepsis (left panel) and in HSCs and myeloid cells in blood and bone marrow obtained from LPS-challenged healthy volunteers (n = 3 for each compartment) at baseline (before LPS administration) and at 4 h post-LPS challenge (right panel). **b**, Overlayed and separated UMAP of HSCs and myeloid cells in peripheral blood and bone marrow obtained from LPS-challenged healthy volunteers (n = 3 for each compartment) at BL, and at 4 h and d7 post-LPS challenge, colored based on compartment (left panels) and acquisition time point (right panels). **c**, Cell type proportion (percentage) of HSC and myeloid lineage cells in peripheral blood and bone marrow obtained from LPS-challenged healthy volunteers (n = 3 for each compartment) at BL and at d7 post-LPS challenge. **d**, Bar plot of log2(relative abundance [percentage] of each cell type) change in HSC and myeloid lineage cells in peripheral blood and bone marrow obtained from LPS-challenged healthy volunteers (n = 3 for each compartment) at BL and at d7 post-LPS challenge. **e**, UMAP of intermediate and non-classical monocytes in HSC and myeloid lineage cells in peripheral blood and bone marrow obtained from LPS-challenged healthy volunteers (n = 3 for each compartment) at baseline and at d7 post-LPS challenge. **f**, UMAP representation of normalized single-cell gene expression of several IFN-I signaling pathway and NCM-enriched genes in HSC and myeloid lineage cells in peripheral blood and bone marrow obtained from LPS-challenged healthy volunteers (n = 3 for each compartment) at baseline and at d7 post-LPS challenge.

To assess the clinical relevance of the LPS systemic inflammation challenge model, we analyzed public scRNA-seq datasets from an early bacterial sepsis cohort which consists of blood samples from patients with sepsis (*n* = 29) and less severe nonseptic bacterial infection (*n* = 17) obtained within 12 h of presentation to the emergency department and healthy controls (*n* = 19)^22^. Furthermore, we analyzed a mixed early COVID-19 or sepsis cohort, which includes blood samples from patients with mild (*n* = 11) and severe or critical (*n* = 12) COVID-19, sepsis (*n* = 13) (all sampled within 7 d of diagnosis) and healthy controls (*n* = 10)^23^. The *LYZ*^+^ monocyte compartment from these two datasets was selected and embedded using UMAP (Fig. 3a,b) and the infMono gene program was projected on to each cohort (Fig. 3c,d). Enrichment analysis indicated a significant enrichment of the infMono gene program in patients with bacterial sepsis and severe COVID-19, who had the highest enrichment scores, as well as in patients with nonseptic bacterial infections, but not in patients with mild COVID-19 (Fig. 3e,f). Conversely, the gene signature induced in the monocytes of patients with sepsis in the early bacterial sepsis cohort^22^, referred to as MS1, was also enriched in the infMono cluster in our LPS-challenged cohort (Extended Data Fig. 7a), indicating the high similarity of early induced genetic programs in bacterial sepsis and LPS-induced systemic inflammation. We also extracted *CD3*^+^ T cells from the early bacterial sepsis and COVID-19 sepsis datasets and embedded them using UMAP (Fig. 3g,h) and projected the inflammatory T cell (T_inf_) cell gene program on these datasets (Fig. 3i,j). Enrichment analysis revealed significantly elevated T_inf_ cell gene program scores across T cells from both datasets, with T cells from patients with COVID-19 exhibiting the highest enrichment (Fig. 3k,l). Thus, the infMono and T_inf_ cell gene programs induced at 4 h post-LPS are significantly elevated during bacterial and COVID-19 sepsis.

### Systemic inflammation impairs myeloid lineage maturation

**Fig. 4:**
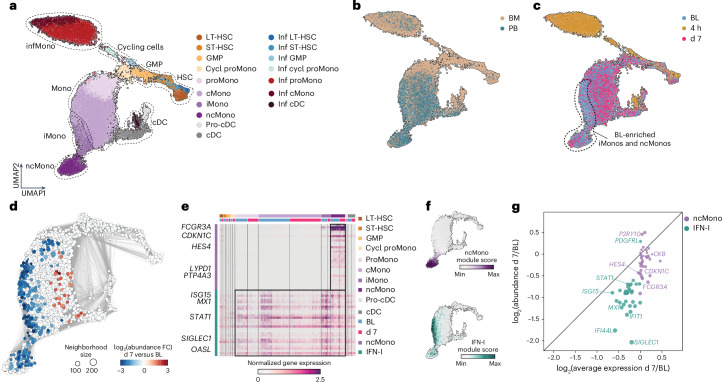
Impairment of monocyte maturation in the late immunosuppressive phase after LPS administration. **a**, UMAP representation of HSCs and myeloid lineage cells from bone marrow and peripheral blood of LPS-challenged healthy volunteers (*n* = 3 for each compartment) at BL (bone marrow, 7 d before the LPS challenge; blood, immediately before the LPS challenge) and at 4 h and d7 post-LPS challenge, colored based on cell type. **b**, UMAP of all blood and bone marrow cells (*n* = 3) as in **a**, colored based on compartment. **c**, UMAP of all blood and bone marrow cells (*n* = 3) as in **a**, colored based on time point. **d**, Nearest neighbor graph-based, differential abundance analysis between BL and d7 of HSCs and myeloid cells from bone marrow and blood of LPS-challenged healthy volunteers (*n* = 3 for each compartment). The blue circles represent significantly reduced neighborhoods on d7. **e**, Heatmap representation of NMF-inferred genes highly expressed in iMonos and ncMonos in cells as in **d** (*n* = 3 for each compartment). **f**, UMAP representation of enrichment of ncMono and IFN-I gene programs in cells as in **d**. **g**, Dot plot representation of log_2_(abundance change) versus log_2_(average expression change) of ncMono and IFN-I gene programs on d7 versus BL in cells as in **d**.

To shed light on the underlying mechanisms responsible for the sustained immunosuppressive effects at d7 post-LPS challenge, we embedded and visualized all HSCs and myeloid cells from all time points (baseline, 4 h and d7 post-LPS) using UMAP (Fig. 4a–c and Extended Data Fig. 7b). Overall, the transcriptome of d7 HSCs and myeloid cells was highly similar to that of baseline HSCs and myeloid cells, with the exception of baseline iMonos and ncMonos (Fig. 4c). Differential abundance analysis using neighboring graphs indicated a significant loss of iMonos and ncMonos at d7 compared with baseline (Fig. 4d and Extended Data Fig. 7c–e), suggesting impaired myeloid maturation. NMF identified two major programs highly active in iMonos and ncMonos (Fig. 4e,f). One comprised genes expressed solely by ncMonos, such as *FCGR3A* and *CDKN1C*, whereas the other comprised IFN-I-responsive genes, such as *IRF7* and *MX1* (Fig. 4e,f and Extended Data Fig. 7f). The average expression per cell for genes associated with the ncMono and IFN-I programs was not significantly altered in *LYZ*^+^ monocytes, but the abundance of cells expressing these two programs was significantly reduced at 7 d compared with baseline (Fig. 4g), suggesting an impairment in myeloid maturation in the immunosuppressive phase of LPS-induced systemic inflammation.

### Sepsis and COVID-19 impair monocyte maturation

**Fig. 5:**
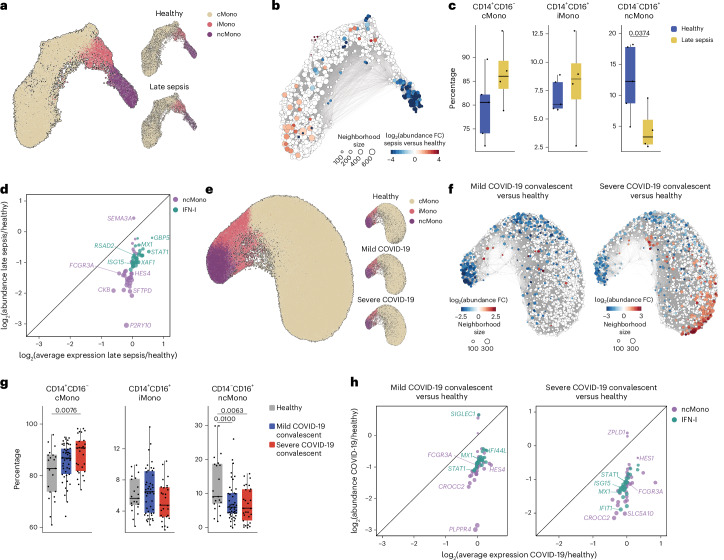
Impaired monocyte maturation in patients with sepsis and COVID-19. **a**, Combined (left) and separated (right) UMAP representations of *LYZ*^+^ blood monocytes from patients with late-stage sepsis (*n* = 4) and healthy controls (*n* = 5), colored based on monocyte subset. **b**, Nearest neighbor graph-based differential abundance analysis between *LYZ*^+^ blood monocytes from healthy participants and patients with late sepsis as in **a**. The blue circles represent significantly reduced neighborhoods in late sepsis versus healthy samples. **c**, Box plots depicting percentages of different monocyte subsets in the blood of patients with late sepsis (*n* = 4) and healthy controls (*n* = 5). **d**, Dot plot representation of log_2_(abundance change) versus log_2_(average expression change) of ncMono-enriched and IFN-I genes in *LYZ*^+^ blood monocytes of patients with late sepsis (*n* = 4) versus healthy controls (*n* = 5). **e**, Combined (left) and separated (right) UMAP representations of *LYZ*^+^ blood monocytes from patients who recovered from mild (*n* = 46) and severe (*n* = 29) COVID-19, as well as healthy controls (*n* = 20), colored based on monocyte subset. **f**, Nearest neighbor graph-based, differential abundance analysis between *LYZ*^+^ blood monocytes from healthy participants and patients who recovered from COVID-19 as in **e**. The blue circles represent significantly reduced neighborhoods in convalescent COVID-19 samples versus healthy ones. **g**, Box plots depicting percentages of different monocyte subsets in the blood of patients who recovered from mild (*n* = 46) and severe (*n* = 29) COVID-19, as well as healthy controls (*n* = 20). **h**, Dot plot representation of log_2_(abundance change) versus log_2_(average expression change) of ncMono-enriched and IFN-I genes in *LYZ*^+^ blood monocytes of blood of patients who recovered from mild (*n* = 46) and severe (*n* = 29) COVID-19 versus healthy controls (*n* = 20). Box plots in **c** and **g** show the median, first and third quartiles and the whiskers 1.5× the IQR. The *P* values were calculated using unpaired, one-sided Wilcoxon’s signed-rank tests.

To test whether impaired monocyte maturation occurred in bacterial sepsis, we leveraged publicly available blood samples from patients with sepsis collected at >14 d into the disease (*n* = 4) and healthy controls (*n* = 5)^24^. All patients had prolonged intensive care unit (ICU) stays and unresolved organ dysfunction^24^. UMAP embedding of *LYZ*^+^ monocytes (Fig. 5a) and nearest neighbor differential abundance analysis unveiled a significant loss of ncMonos in patients with late sepsis compared with healthy controls (Fig. 5b,c). Genes related to the ncMono and IFN-I gene programs showed minor changes in average gene expression per cell, whereas the abundance of cells that expressed these programs, especially the ncMono program, was significantly reduced in patients with late sepsis compared with healthy controls (Fig. 5d).

We also leveraged a public scRNA-seq dataset of patients who recovered from mild (*n* = 46) and severe (*n* = 29) COVID-19 as well as healthy controls (*n* = 20)^25^. Blood samples from patients were collected >14 d after the onset of symptoms to evaluate the late or convalescent phase of the disease. UMAP embedding of *LYZ*^+^ monocytes (Fig. 5e) and nearest neighbor differential abundance analysis showed a significant loss of ncMonos in patients who were convalescing from COVID-19 compared with healthy controls, both in patients who previously suffered mild and severe COVID-19 (Fig. 5f,g), with no significant changes in the average expression per cell of genes related to ncMono and IFN-I gene programs, but rather a reduction in the abundance of cells that expressed these programs (Fig. 5h).

Our observations revealed a significant reduction in the number of ncMonos in the late phase of bacterial sepsis or COVID-19, implying an impairment in the monocyte maturation process in these conditions.

### IFNβ reverses immunosuppression and promotes monocyte maturation

**Extended Data Fig. 8:**
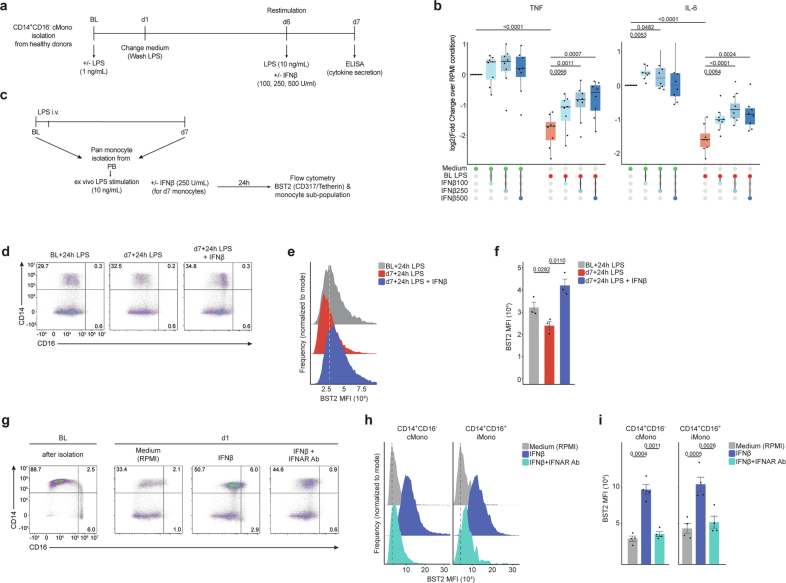
IFNβ treatment reverses LPS-induced immunosuppression and promotes monocyte maturation. **a**, Schematic representation of *in vitro* reversal experiments with IFNβ in LPS-immunosuppressed CD14^+^CD16^−^ classical monocytes (cMonos). **b**, Box plots of TNF (left panel) and IL-6 (right panel) production by cMonos obtained from healthy donors (n = 8) that were incubated in the presence or absence of 1 ng mL^−1^ LPS for 24 h and restimulated after 6 d with 10 ng mL^−1^ LPS in the presence or absence of various concentration of IFNβ (100, 250, 500 U mL^−1^). The log2fold changes are over medium control (RPMI)-cultured cells that were stimulated with LPS on day 6. **c**, schematic representation of *ex vivo* BST2 flow cytometry experiments in immunosuppressed pan monocytes (containing all subsets) **d**, Representative example of flow cytometric analysis of monocyte subsets in Cmonos obtained at baseline and at d7 post-LPS that were *ex vivo* stimulated for 24 h with LPS (10 ng mL^−1^) in the presence and absence of IFNβ (250 U mL^−1^). **e**, Overlay of BST2 mean fluorescence intensity (MFI) in Cmonos obtained at baseline and at d7 post-LPS that were *ex vivo* stimulated for 24 h with LPS (10 ng mL^−1^) in the presence and absence of IFNβ (250 U mL^−1^). **f**, Bar plot of BST2 MFI in cMonos (n = 3) obtained at baseline and at d7 after 24 h of *ex vivo* stimulation with LPS (10 ng mL^−1^) in the presence and absence of IFNβ (250 U mL^−1^). **g**, Representative flow cytometry profiles of monocyte subsets in pan monocytes obtained from healthy donors (n = 4) immediately after isolation and following 24 h of incubation with or without IFNβ (250 U mL^−1^) in the presence and absence of IFNAR antibody (IFNAR Ab, 10 μg mL^−1^). **h**, Overlay of BST2 MFI on cMonos and CD14^+^CD16^+^ intermediate monocytes (iMonos) following 24 h of incubation with or without IFNβ (250 U mL^−1^) in the presence and absence of IFNAR Ab (10 μg mL^−1^). **i**, Bar plot of BST2 MFI on cMonos and iMonos following 24 h of incubation with or without IFNβ (250 U mL^−1^) in the presence and absence of IFNAR Ab (10 μg mL^−1^). Box plots in **b** show median, first and third quartiles; whiskers show 1.5x the interquartile range. Bar plots in **f**, **i** presented as mean values +/− SEM. The *P* values were calculated using paired two-sided t-tests.

To test whether IFNβ, a type-I IFN, could reverse LPS-induced immunosuppression, in vitro blood cMonos obtained from healthy donors (*n* = 8) were incubated in the presence or absence of 1 ng ml^−1^ of LPS for 24 h, and restimulated after 6 d with 10 ng ml^−1^ of LPS in the presence or absence of various concentration of IFNβ (100, 250, 500 U ml^−1^). TNF and IL-6 production by cMonos that had previously been exposed to LPS was significantly reduced compared with cMonos that had not been exposed to LPS (Extended Data Fig. 8a,b). All concentrations of IFNβ restored the production of TNF and IL-6 in cMonos that had been previously exposed to LPS, whereas production of TNF and IL-6 in cMonos not previously exposed to LPS was less affected (Extended Data Fig. 8b).

**Fig. 6:**
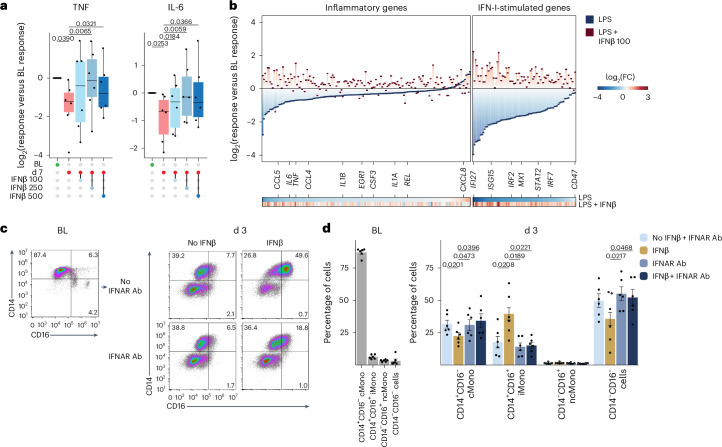
IFNβ treatment reverses LPS-induced immunosuppression. **a**, Box plots of log_2_(fold-change) in TNF (left) and IL-6 (right) production by cMonos obtained from LPS-challenged volunteers (*n* = 6) on BL and d7 who were stimulated with LPS (10 ng ml^−1^) in the presence or absence of IFNβ (100, 250, 500 U ml^−1^) for 24 h. **b**, Comparison of expression of inflammatory and ISGs between cMonos obtained from LPS-challenged volunteers (*n* = 3) at BL and d7 who were stimulated with 10 ng ml^−1^ of LPS in the presence or absence of IFNβ (100 U ml^−1^) for 4 h. Data were normalized to the response of BL cMonos. **c**, Flow plots of a representative example of CD14 and CD16 expression of cMonos obtained from a healthy donor immediately after isolation (BL) and after 3 d of incubation with or without 100 U ml^−1^ of IFNβ in the presence or absence of an anti-IFNAR Ab (10 μg ml^−1^). The experiment was repeated in six donors (see **d**). **d**, Bar plots of percentages of monocyte subsets in the blood of healthy donors (*n* = 6) immediately after cMono isolation (BL) and at d3 of incubation of cMonos with or without 100 U ml^−1^ of IFNβ in the presence or absence of IFNAR Ab (10 μg ml^−1^). The box plots in **a** show the median and first and third quartiles and the whiskers 1.5× the IQR. Bar plots in **d** are presented as mean values ± s.e.m. The *P* values were calculated using two-sided, paired Student’s *t*-tests.

Subsequently, we evaluated whether IFNβ could reverse the impaired cytokine production by monocytes observed at d7 post-in vivo LPS administration. To explore this, blood cMonos obtained from LPS-challenged healthy volunteers at baseline and d7 were stimulated ex vivo with 10 ng ml^−1^ of LPS in the presence or absence of IFNβ (100, 250, 500 U ml^−1^) and analyzed at 4 h using RNA-seq (*n* = 3, 100 U ml^−1^ of IFNβ only) and at 24 h for TNF and IL-6 production (*n* = 6), as well as BST expression (*n* = 3, 250 U ml^−1^ of IFNβ only). TNF and IL-6 production by cMonos obtained at d7 was significantly reduced compared with production by cMonos obtained at baseline (Fig. 6a). All concentrations of IFNβ markedly enhanced the LPS-induced production of TNF and IL-6 in cMonos obtained at d7 post-LPS (Fig. 6a). Analysis of RNA-seq profiles indicated that IFNβ treatment strongly upregulated proinflammatory genes (for example, *TNFSF10*, *IL6*, *TNF* and *IL1B*) and ISGs (for example, *IFI27*, *ISG15*, *IRF7* and *MX1*) in LPS-stimulated cMonos obtained at d7 (Fig. 6b). Flow cytometry confirmed restoration of IFN-I responses by IFNβ, because the reduced cell surface expression of BST2 on LPS-stimulated cMonos obtained at d7 post-LPS compared with cMonos obtained at baseline was abrogated after IFNβ treatment (Extended Data Fig. 8c–f).

To investigate whether IFNβ modulated monocyte maturation, blood cMonos obtained from healthy donors (*n* = 6) were treated with or without 100 U ml^−1^ of IFNβ in the presence or absence of an IFN-I receptor (IFNAR) antibody (IFNAR Ab, 10 μg ml^−1^) for 3 d. We detected an increased abundance of iMonos in IFNβ-treated cultures compared with untreated cultures, an effect reversed by the IFNAR antibody (Fig. 6c,d). We did not observe an effect of IFNβ on the frequency of ncMonos (Fig. 6c,d), suggesting that additional factors are required for maturation to ncMonos or monocytes do not mature into ncMonos in these in vitro conditions. Culturing of blood pan-monocytes obtained from healthy donors (*n* = 4), with or without 250 U ml^−1^ of IFNβ in the presence and absence of IFNAR antibody (10 μg ml^−1^) for 24 h, resulted in the loss of ncMonos in all conditions (Extended Data Fig. 8g), whereas IFNβ treatment significantly increased the expression of BST2 on cMonos and iMonos, compared with untreated cultures, and the IFNAR antibody reversed this effect (Extended Data Fig. 8h,i).

## Discussion

In this paper, we have described the dynamic transcriptional and functional landscape of peripheral blood and bone marrow leukocytes during the acute hyperinflammatory and late immunosuppressive phases induced by LPS administration in healthy volunteers. In the acute hyperinflammatory phase, distinct subsets of monocyte-like cells (*CD163*^+^*SLC39A8*^+^*CALR*^+^ infMonos) and T_inf_ cells (*MX1*^+^*IRF1*^+^*ISG15*^+^) emerged, a phenomenon also observed in patients with sepsis and COVID-19. The late immunosuppressive phase was characterized by a severely suppressed in vivo cytokine response to a second challenge with the same dose of LPS, ex vivo hyporesponsiveness of monocytes to a variety of inflammatory stimuli, suppressed IFN-I signaling and impaired myelopoiesis resulting in decreased maturation of monocytes toward iMonos and ncMonos. The impaired monocyte maturation was also observed in the late phase of sepsis or COVID-19.

The infMonos that emerged 4 h after LPS administration exhibited upregulated expression of inflammatory genes (for example, *CD163*, *SLC39A8* and *CALR*) and immature pro-monocyte markers such as *RETN*, indicative of emergency myelopoiesis. This bears a similarity to the induction of immature MDSCs observed in patients with sepsis^22,26^. The acute hyperinflammatory phase was also characterized by activation of the IFN-I signaling pathway across multiple cell types, including HSCs, consistent with reports that LPS induces IFNβ and ISG expression^27,28^. IFN-I induction in murine HSCs can activate HSCs to proliferate and thereby contribute to emergency myelopoiesis, while inducing HSC exhaustion in the long term^29–31^.

Seven days after the LPS challenge, in the late immunosuppressive phase, the in vivo response to a secondary challenge with the same dose was severely suppressed. This immunosuppression is unlikely to be caused by direct exposition of (pro)monocytes to LPS because of the relatively short lifespan of monocytes (~1–2 d)^16^, their rapid disappearance from the circulation after LPS administration^16^ and the swift clearance of LPS from the blood (half-life ~30 min)^32,33^. Therefore, it is plausible that the immunosuppressed monocytes originate from stem or progenitor cells in the bone marrow exposed to systemic inflammation.

A significant reduction in the abundance of iMonos and ncMonos was observed in the late immunosuppressive phase, suggesting a maturation impairment. As iMonos and ncMonos were the highest ISG-expressing cells and IFNβ treatment resulted in maturation toward iMonos, our results imply an important role for IFN-I signaling in monocyte maturation. A possible causal link between diminished IFN-I response and impaired myelopoiesis is substantiated by findings in mice deficient for either IFNAR1 or IFNβ that generate significantly less mature monocytes, which express fewer ISGs^34,35^.

The infMono and T_inf_ cell gene programs identified during the acute phase of LPS-induced systemic inflammation were also significantly induced during the acute hyperinflammatory phase of bacterial sepsis and COVID-19. Furthermore, we detected a significant loss of ncMonos in patients with late-phase sepsis and individuals convalescing from COVID-19, akin to what we observed at d7 after LPS administration. These observations indicate that the LPS-induced systemic inflammation model recapitulated hallmark features of highly heterogeneous clinical complications, such as sepsis, rendering it a suitable in vivo model to both interrogate the pathophysiology of inflammatory diseases and evaluate the efficacy of new therapeutic interventions.

The impairment in IFN-I signaling during the late immunosuppressive phase was multifaceted, involving the aforementioned reduction in the number of ncMonos, which were high expressors of IFN-I genes, but also significantly impaired ISG induction in cMonos upon ex vivo restimulation with LPS. This underscores the complexity of immunosuppression in systemic inflammatory conditions and highlights the need for targeted interventions that can both restore monocyte maturation and reactivate IFN-I signaling pathways. IFNβ reversed the immunosuppressed state of monocytes by reinstating the expression of inflammatory genes, ISGs and cytokine production, and induced maturation of cMonos toward iMonos.

A limitation of the present study is the use of a male-only study cohort, which limits the ability to apply these findings to female patients. Females make significantly more proinflammatory cytokines in response to LPS administration than males^36,37^. However, despite the sex-based differences in the magnitude of the response, the molecular mechanisms underlying the immune response to LPS are highly conserved and there are no significant differences in the immune-related pathways enriched in both sexes^36^, so this model of systemic inflammation can guide future research in more complex disease models or clinical scenarios in both females and males.

Overall, our findings highlight a critical role of diminished IFN-I signaling in impaired myelopoiesis and immunosuppression and reveal that IFNβ may represent a promising treatment option to reverse immunosuppression in systemic inflammatory conditions.

## Methods

### Participants and ethical approval

The LPS-induced systemic inflammation (experimental human endotoxemia) study was approved by the Medical Ethical Committee Oost-Nederland (ref. no. NL61136.091.17). Eleven healthy male volunteers were recruited. All participants gave written informed consent and medical history, physical examination and laboratory tests and a 12-lead electrocardiogram did not reveal any abnormalities. Smoking, medication use, previous participation in experimental human endotoxemia studies or signs of acute illness within 3 weeks before the start of the study were exclusion criteria. For in vitro experiments, blood was drawn from healthy volunteers after obtaining written informed consent. Blood withdrawal for this purpose was approved by the Medical Ethical Committee Oost-Nederland (ref. no. NL84281.091.23). All study procedures were performed in accordance with the Declaration of Helsinki, including the latest revisions.

### LPS-induced systemic inflammation study design and procedures

We performed a randomized, placebo-controlled observational study in which participants were allocated to receive either an intravenous LPS injection (*n* = 7) or a placebo injection with 0.9% NaCl (*n* = 4). All LPS-challenged participants received a second LPS challenge on d7 after the first LPS challenge, using identical procedures. Bone marrow and blood were collected at baseline (bone marrow: day −7 compared with first LPS challenge, blood: immediately before the first LPS challenge), 4 h after the first LPS challenge (4 h) and at d7. Bone marrow aspiration from the posterior iliac crest was performed by a skilled physician assistant of the Department of Hematology at Radboud university medical center (Radboudumc) in Nijmegen, the Netherlands. Bone marrow was collected in a sodium heparin solution (150 IU ml^−1^, ratio 3:1). Blood was collected in tubes containing EDTA as an anticoagulant. Additional blood was collected before and at several time points after the LPS challenges.

During LPS or placebo challenge days, all participants underwent the same study procedures, except for administration of either LPS or placebo. Briefly, 24 h before hospitalization, participants needed to refrain from alcohol and caffeine and from 22:00 onward no food and drinks were allowed. Before the challenge, participants were admitted to the research ICU of Radboudumc. An intravenous cannula was placed in an antebrachial vein to administrate fluids and LPS or 0.9% NaCl. A radial artery catheter was inserted to withdraw blood and monitor blood pressure continuously. Prehydration (1.5 l of 2.5% glucose/0.45% NaCl) was administered intravenously in the hour before the challenge. Thereafter, a bolus of 2 ng kg^−1^ of LPS (*E. coli* type O113, List Biological Laboratories, lot no. 94332B1) or saline (placebo) was administered intravenously and hydration fluid (2.5% glucose/0.45% NaCl) was continued at an infusion rate of 150 ml h^−1^ for 8 h. During hospitalization (up to 8 h post-LPS administration), the heart rate was monitored using a four-lead electrocardiogram (M50 Monitor, Philips), core temperature was measured at 30-min intervals with a tympanic thermometer (FirstTemp Genius 2, Covidien) and LPS-induced symptoms (headache, nausea, cold shivers and muscle and back pain) were scored using a numerical six-point scale (0 = no symptoms, 5 = worst symptoms experienced ever) with the addition of 3 points in case of vomiting, resulting in a total symptom score ranging from 0 to 28.

### Cell counts and plasma cytokine measurements

Blood cell counts were analyzed using a Sysmex XE-5000. For cytokine determination, blood was centrifuged directly after withdrawal (10 min, 2,000*g*, 4 °C) and plasma was stored at −80 °C until analysis. Concentrations of TNF, IL-6, CXCL8 (IL-8), IL-10, CCL3 (MIP-1α), CCL2 (MCP-1), CCL4 (MIP-1β) and IL-1RN (IL-1RA) were determined in one batch using a simultaneous Luminex assay (Milliplex, Millipore) on a MagPix instrument (Luminex).

### Flow cytometry of monocyte subsets

**Extended Data Fig. 9:**
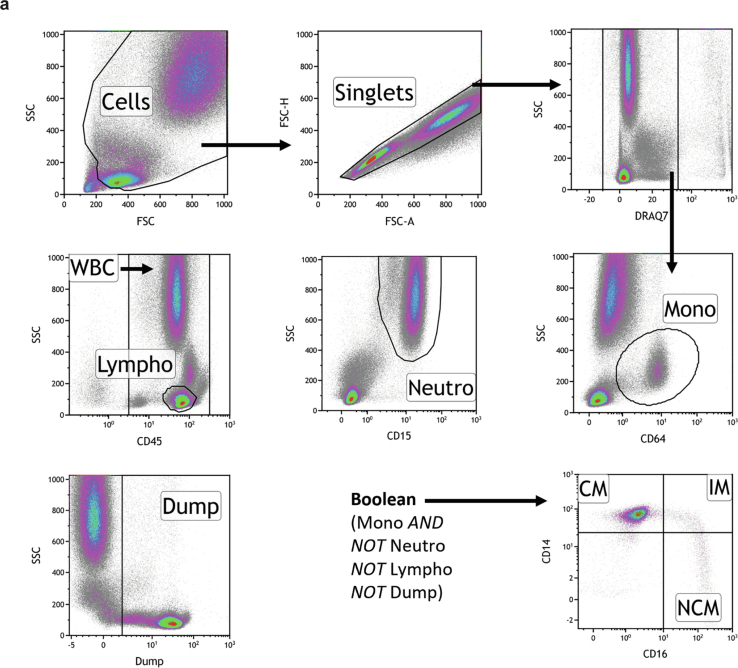
Gating strategy to analyze monocyte subsets in peripheral blood. **a**, Gating strategy used to identify three monocyte subsets in peripheral blood of LPS-challenged volunteers by flow cytometry. CM: classical monocyte (cMono); IM: intermediate monocyte (iMono); NCM: non classical monocyte (ncMono).

Blood from LPS-challenged healthy volunteers was phenotyped with antibodies against CD45-Cy5.5 (Beckman Coulter, cat. no. A62835), CD14-ECD (Beckman Coulter, cat. no. B92391), CD16-PE (BD Biosciences, cat. no. 332779), CD64-FITC (Beckman Coulter, cat. no. B49185), CD11b-PC7 (Beckman Coulter, cat. no. A54822), HLA-DR-APC (Beckman Coulter, cat. no. IM3635), DRAQ7 (Biostatus, cat. no. DR71000), CD192-BV421 (BD Biosciences, cat. no. 564067), CD15-KO (Beckman Coulter, cat. no. B01176) and dumpgates CD3-AA750 (Beckman Coulter, cat. no. A94680), CD19-APC A750 (Beckman Coulter, cat. no. A94681) and CD56-APC A750 (Beckman Coulter, cat. no. B46024) on a Navios flow cytometer (Beckman Coulter) at the Department of Hematology of Radboud UMC. Monocyte subtype populations were determined using the gating strategy depicted in Extended Data Fig. 9a. We used 1:100 dilution for all antibodies used in the present study.

### In vitro, ex vivo and stimulation experiments

#### Ex vivo stimulation of monocytes for cytokine production

Classical monocytes (CD14^+^CD16^−^) were isolated from participants taking part in the experimental endotoxemia study at baseline (before LPS administration, d0) as well as at 4 h and 7 d after in vivo LPS administration. To this aim, peripheral blood mononuclear cells (PBMCs) were isolated using Ficoll-based density gradient separation (1,200*g*, 10 min, room temperature, with brake) in SepMate-50 tubes (STEMCELL Technologies). All samples were kept on ice in between procedures. PBMCs were subsequently depleted from neutrophils and intermediate and nonclassic monocytes using CD16 microbeads (Miltenyi Biotec) according to the manufacturer’s protocol. Hereafter, classic monocytes were positively selected using CD14 microbeads (Miltenyi Biotec). Cells were resuspended in culture medium (Roswell Park Memorial Institute (RPMI)-1640, Dutch modification, supplemented with 50 µg ml^−1^ of gentamicin, 1 mM sodium pyruvate and 2 mM GlutaMAX), and 1 × 10^5^ cells were seeded in flat-bottomed 96-well plates. The cells were incubated with culture medium or various stimuli (Pam3Cys (10 µg ml^−1^; InvivoGen), poly(I:C) (50 µg ml^−1^; InvivoGen), *E. coli* LPS (10 ng ml^−1^, Sigma-Aldrich, serotype 055:B5), flagellin (10 µg ml^−1^, InvivoGen), resiquimod (R848, 0.35 µg ml^−1^, InvivoGen), heat-killed *E. coli* (10^7^ per well, ATCC35218), *S. aureus* (10^7^ per well, American Type Culture Collection (ATCC), cat. no. 25923), *P. aeruginosa* (10^7^ per well, PA01), *C. albicans* (10^6^ per well, UC820) and *Aspergillus fumigatus* (10^7^ per well (with 10% human pooled serum), V05-27)) for 24 h. Hereafter, supernatants were collected and stored at −80 °C until analysis. The concentrations of TNF, IL-1β, IL-1RN (IL-1RA), IL-6, IL-10 and CCL4 (MIP-1β) in supernatants of ex vivo stimulated cell cultures were determined in one batch using a simultaneous Luminex assay on a MagPix instrument (Luminex).

#### Ex vivo stimulation of monocytes for analysis of BST2 expression

Pan-monocytes (containing all subsets) were isolated from participants (*n* = 3) in the experimental endotoxemia study at baseline (before LPS administration d0) as well as at 7 d after in vivo LPS administration. To this end, cryopreserved PBMCs (isolated as described above) were thawed, and monocytes were isolated using a human pan-monocyte isolation kit (negative selection, Miltenyi Biotec). Cells were resuspended in culture medium (RPMI-1640, Dutch modification, supplemented with 50 µg ml^−1^ of gentamicin, 1 mM sodium pyruvate and 2 mM GlutaMAX). Subsequently, 1 × 10^5^ cells were seeded in round-bottomed, poly(propylene) 96-well plates and incubated in the presence and absence of *E. coli* LPS (10 ng ml^−1^) and/or IFNβ (250 U ml^−1^, R&D Systems) for 24 h, after which samples were stained and analyzed using flow cytometry (Cytoflex, Beckman Coulter). Flow cytometry antibodies used were mouse anti-human CD45-allophyocyanin (APC)/A750, mouse anti-human CD14-APC antibody (Beckman Coulter), mouse anti-human CD16-phycoerythrin (PE)/Cy7 antibody (BD Biosciences) and mouse anti-human BST2 (CD137/Tetherin) PE (Beckman Coulter).

#### Isolation and ex vivo stimulation of monocytes for bulk RNA-seq

PBMCs were isolated from participants in the experimental endotoxemia study at baseline (before LPS administration, 0 h) and at 4 h, 8 h, 24 h and 7 d and 7 d + 4 h after in vivo LPS administration. PBMCs were isolated using Ficoll-based density gradient separation (1,200*g*, 10 min, room temperature, with brake) in SepMate-50 tubes. Cells were washed with cold phosphate-buffered saline (PBS) (1,700 rpm, 10 min, 4 °C) and CD14^+^ monocytes were isolated by positive selection using CD14 microbeads (Miltenyi Biotec). Unstimulated cells were lysed using TRIzol (Invitrogen) and stored at −80 °C until preparation for bulk RNA-seq analysis (described separately below). For ex vivo stimulation, cells were resuspended in culture medium (RPMI-1640, Dutch modification, supplemented with 50 µg ml^−1^ of gentamicin, 1 mM sodium pyruvate, 2 mM GlutaMAX and 10% human serum) and seeded in flat-bottomed, 96-well plates (2 × 10^5^ cells per well). Cells were left to attach for 1 h, after which culture medium was refreshed and monocytes were (re)stimulated with 10 ng ml^−1^ of LPS (Sigma-Aldrich) for 4 h. After stimulation, monocytes were lysed using TRIzol and stored at −80 °C until preparation for bulk RNA-seq analysis.

#### Co-culture experiments with monocytes and T cells

PBMCs of nine healthy donors were isolated using Ficoll-based density gradient separation (1,200*g*, 10 min, room temperatures, with brake) in SepMate-TM-50 tubes. Cells were washed with cold PBS (1,700 rpm, 10 min, 4 °C), resuspended in culture medium (RPMI-1640, Dutch modification, Gibco, supplemented with 50 µg ml^−1^ of gentamicin, 1 mM sodium pyruvate and 2 mM GlutaMAX) and counted on a Sysmex XN-450 (Sysmex Nederland). To obtain a pure lymphocyte fraction, PBMCs were depleted of monocytes using EasySep Human CD14 Positive Selection kit II (STEMCELL Technologies) in a 96-well, non-TC-treated, round-bottomed plate (Corning) in combination with the EasyPlate EasySep Magnet (STEMCELL Technologies). Lymphocytes were counted on a Sysmex XN-450 and brought to a concentration of 1 × 10^6^ ml^−1^ in PBS for carboxyfluorescein succinimidyl ester (CFSE) labeling using the CellTrace CFSE Cell Proliferation kit (Thermo Fisher Scientific). Lymphocytes were labeled with 500 nM CFSE for 5 min at 37 °C with gentle agitation in the dark. After incubation, ice-cold fetal bovine serum (FBS) Xtra (Capricorn Scientific) was added in a 1:1 ratio to quench any remaining dye. CFSE-labeled lymphocytes were washed once with RPMI-1640, Dutch modification, supplemented with 10% FBS Xtra. After washing, lymphocytes were resuspended in 1 ml of RPMI-1640, Dutch modification containing 10% bovine calf serum (BCS, Capricorn Scientific) and counted on a Sysmex XN-450. Lymphocytes were subsequently kept on ice until cryopreserved classic monocytes (CD14^+^CD16^−^) obtained from participants in the experimental endotoxemia study were thawed. These classic monocytes were obtained from PBMCs isolated at baseline (before LPS administration, 0 h) and at 4 h after in vivo LPS administration, as described above. For co-culture experiments, cells were thawed and washed using pre-warmed (37 °C) complete medium (RPMI-1640, Dutch modification, supplemented with 20% FBS Xtra and 100 µg ml^−1^ of DNase 1). Monocytes were then resuspended in ice-cold RPMI-1640, Dutch modification, containing 10% BCS and counted on Sysmex XN-450. Subsequently, 5 × 10^4^ CFSE-labeled lymphocytes were cultured in the presence and absence of 1 × 10^5^ monocytes (1:2 lymphocyte:monocyte ratio) in a 96-well, TC-treated, round-bottomed plate (Sarstedt). To activate lymphocyte proliferation and cytokine production, 5 × 10^4^ CD3/CD28 Dynabeads were added to the wells (Thermo Fisher Scientific) and lymphocyte or monocyte co-cultures were left to incubate for 3 d. Thereafter, supernatants were stored at −20 °C for analysis of TNF and IFNγ production using ELISA (R&D Systems), whereas T cell proliferation was analyzed using flow cytometry (Cytoflex, Beckman Coulter) after staining of cells with a mouse anti-human CD3-PeC7 antibody (Beckman Coulter).

#### In vitro reversal of immunosuppression using IFNβ

Classic monocytes (CD14^+^CD16^−^) of eight healthy donors were isolated as described above. Cells were resuspended in culture medium (RPMI-1640, Dutch modification, supplemented with 50 µg ml^−1^ of gentamicin, 1 mM sodium pyruvate and 2 mM GlutaMAX), and 1 × 10^5^ cells per well were seeded in flat-bottomed, 96-well plates. Cells were left to attach for 1 h, after which culture medium was refreshed with culture medium supplemented with 10% human serum. Cells were then incubated for 24 h in the presence or absence of 1 ng ml^−1^ of LPS. After 24 h, cells were washed with warm PBS. Thereafter, cells were incubated with culture medium supplemented with 10% human serum for 5 d and this culture medium was refreshed on d3. On d6, culture medium was removed and cells were incubated for 24 h in culture medium with 10 ng ml^−1^ of LPS in the presence and absence of IFNβ (100, 250 and 500 U ml^−1^, R&D Systems) for 24 h. Afterwards, supernatants were collected and stored at −80 °C until determination of TNF and IL-6 using ELISA (R&D Systems).

#### Ex vivo reversal of immunosuppression using IFNβ

Cryopreserved classic monocytes (CD14^+^CD16^−^, isolated as described above) obtained from participants in the experimental endotoxemia study at baseline (before LPS administration, d0) and 7 d after in vivo LPS administration were thawed, washed (see above) and resuspended in culture medium (RPMI-1640, Dutch modification, supplemented with 50 µg ml^−1^ of gentamicin, 1 mM sodium pyruvate, 2 mM GlutaMAX and 10% human serum). Subsequently, 3 × 10^5^ monocytes were stimulated with 10 ng ml^−1^ of LPS in the presence or absence of IFNβ (100, 250 and 500 U ml^−1^) for 4 and 24 h in a 96-well, TC-treated, flat-bottomed plate. After 4 h, cells were stored in RLT buffer containing 40 mM dithiothreitol (QIAGEN) for future RNA isolation and, after 24 h, supernatants were stored at −80 °C until determination of TNF and IL-6 using ELISA.

#### In vitro monocyte maturation using IFNβ and responsiveness across monocyte subsets

Maturation experiments: classic monocytes (CD14^+^CD16^−^) of six healthy donors were isolated as described above. Monocytes were subsequently incubated in RPMI-1640, Dutch modification, supplemented with 50 µg ml^−1^ of gentamicin, 1 mM sodium pyruvate, 2 mM GlutaMAX and 50% human serum for 30 min on ice to block unwanted antibody FC receptor binding. Thereafter, 2.5 × 10^5^ monocytes were incubated for 1 h in 5-ml Falcon, poly(propylene), round-bottomed tubes (Corning) with culture medium (RPMI-1640, Dutch modification, supplemented with 50 µg ml^−1^ of gentamicin, 1 mM sodium pyruvate, 2 mM GlutaMAX and 10% human serum) in the presence or absence of 10 µg ml^−1^ of mouse anti-human IFNα/-β receptor chain 2 antibody (Merck Life Science). Next, 100 U ml^−1^ of IFNβ (R&D Systems) or culture medium was added and cells were incubated for 3 d, after which they were stained and analyzed using flow cytometry (Cytoflex, Beckman Coulter). Flow cytometry antibodies used were mouse anti-human CD45-APC/A750, mouse anti-human CD14-APC antibody (Beckman Coulter) and mouse anti-human CD16-PE/Cy7 antibody (BD Biosciences).

Responsiveness to IFNβ across subsets: pan-monocytes (containing all subsets) of four healthy donors were isolated as described above. Cells were resuspended in culture medium (RPMI-1640, Dutch modification, supplemented with 50 µg ml^−1^ of gentamicin, 1 mM sodium pyruvate, 2 mM GlutaMAX and and 50% human serum) was added for 30 min on ice to block unwanted antibody FC receptor binding. Subsequently, 1 × 10^5^ cells were seeded in round-bottomed, poly(propylene), 96-well plates and incubated in the presence and absence of 10 µg ml^−1^ of mouse anti-human IFNα/-β receptor chain 2 antibody for 1 h. Subsequently cells were incubated with or without of *E. coli* LPS (10 ng ml^−1^, Sigma-Aldrich, serotype 055:B5) and IFNβ (100 U ml^−1^, R&D Systems) for 24 h, after which samples were stained and analyzed using flow cytometry. The flow cytometry antibodies used are described above.

### Bulk RNA-seq

#### Total RNA extraction and cDNA synthesis

Total RNA was extracted from monocytes using the RNeasy RNA extraction kit (QIAGEN), incorporating on-column DNase I (QIAGEN) DNA digestion. Afterwards, ribosomal RNA was removed using riboZero rRNA removal kit (Illumina). The efficiency of rRNA removal was confirmed using a reverse transcription quantitative PCR (RT–qPCR) with primers for glyceraldehyde 3-phosphate dehydrogenase (as internal control) and 18S and 28S rRNA. RNA molecules fragmented into ~200-bp fragments by incubating in fragmentation buffer (200 mM Tris acetate, 500 mM potassium acetate and 150 mM magnesium acetate, pH 8.2) for 7.5 min at 95 °C. First-strand complementary DNA from fragmented RNA was synthesized using SuperScript III reverse transcriptase enzyme (Life Technologies) according to the manufacturer’s protocol and followed by second-strand cDNA synthesis.

#### Bulk RNA-seq library preparation and sequencing

Gene expression libraries were prepared using KAPA HyperPrep kit (KAPA Biosystems) according to the manufacturer’s protocol. In brief, synthesized double-stranded cDNA was incubated with end repair and A-tailing buffer and enzyme initially for 30 min at 20 °C and then for 30 min at 65 °C. Library-specific adapters were ligated to tailed DNA molecules using DNA ligase enzyme by incubating for 15 min at 15 °C. Ligation reaction was cleaned up using Agencourt AMPure XP reagent (Beckman Coulter) and subsequently amplified using ten cycles of PCR. Finally, 300-bp fragments were selected using a 2% E-gel selection system (Invitrogen). Size selection was validated with a 2100 BioAnalyzer system (Agilent). Prepared libraries were sequenced utilizing NextSeq 500 machine (Illumina) with a paired-end sequencing setup.

#### Bulk RNA-seq data analysis

Raw RNA-seq reads were aligned to the hg38 reference genome and gene expression profiles were quantified using STAR aligner^38^. Genes with <50 mapped reads on condition average were excluded from the analysis. For each comparison of RNA-seq profiles, gene expression data of corresponding samples was normalized and differentially expressed genes (DEGs) were identified using the DESeq2 (ref. ^39^) analysis package utilizing fold-change >2 and *q* value (Benjamini–Hochberg-adjusted *P* (*P*_adj_) value) <0.05 as statistical significance cutoffs.

### GO and GSEA of bulk RNA-seq data

To infer significantly enriched gene ontologies (GOs) for identified gene sets of interest, such as DEGs, we used the clusterProfiler^40^ analysis package. Gene set enrichment analysis (GSEA) for the comparison of d0 and d7 monocyte gene expression profiles was done using the fgsea^41^ package.

### ScRNA-seq sample preparation and sequencing

For scRNA-seq, mononuclear cells (MNCs) were isolated from peripheral blood and bone marrow samples using Ficoll-based density gradient separation (1,200*g*, 10 min, room temperature, with brake) in SepMate-50 tubes, as described above, and cryopreserved at −80 °C until further processing. No depletion or selection of a specific myeloid MNC was performed and the profiling was unbiased. Single Cell Gene Expression 3ʹ v.3 (10x Genomics) was utilized following the manufacturer’s protocol. In brief, approximately 10,000–15,000 single cells were loaded into a channel of chromium chip and the loaded chip was inserted into the chromium controller. After generation of single-cell gel bead-in-emulsion and reverse transcription of RNA, cDNA amplification, fragmentation and adapter ligation were done. The quality of prepared sequencing libraries was assessed using 2100 Bioanalyzer (Agilent). The scRNA-seq libraries were sequenced on a NextSeq 500 (Illumina) or NovaSeq (Illumina).

### ScRNA-seq data preprocessing and analysis

Demultiplexing the raw BCL files was done using Cell Ranger mkfastq (v.3.1.0) software and the resulting fastq files were mapped to the human GRCh38 reference genome using Cell Ranger count software with default parameters. The output count matrix was imported to R analysis software and further analyzed using Seurat (v.4.0.4)^42^. Low-quality cells with mitochondrial percentage >15% or with <200 genes and <40,000 UMI (unique molecular modifier) counts were excluded from the analysis. Cells expressing multi-canonical lineage markers at the same time, such as T and B cell-specific markers, were identified as potential doublets and removed from the analysis. Afterwards, gene expression profiles were normalized to sequencing depth and scaled to 10,000 counts and log(transformed). Batch correction for interdonor differences was done utilizing the RunFastMNN function from the SeuratWrappers package, which is the R implementation of the MNN^43^ batch correction method. Cells were embedded on a two-dimensional view using UMAP^19^ and clustered using the FindClusters function from the Seurat package with a resolution of 1. Cluster- or cell-type-specific marker genes were identified using FindMarkers function from the Seurat package with default parameters. Based on well-known cell-type markers, we annotated each cluster and visualized violin plots of gene expression for several cell-type-specific markers using the stacked_violin function of the scanpy (1.9.1)^44^ analysis package in Python. Cell density plots were generated using the embedding_density function of the scanpy package. For lineage-specific analysis, the same steps were done by initial extract of corresponding lineage cells from the whole bone marrow dataset and further embedding and clustering. Unless specified, all figures were generated using the ggplot2 visualization package and all heatmaps were generated using pheatmap package in R.

### Statistical differential abundance analysis

To perform differential abundance analysis and find out significantly reduced (or induced) populations we used the miloR package^45^. This package uses the *k*-nearest neighbor graph base differential abundance analysis, taking into account the metadata from different donors for each condition. We used the suggested workflow of the miloR package for the analysis, setting the *k* parameter to 30 and the prop parameter to 0.1 for LPS systemic inflammation and late sepsis datasets and 0.05 for the COVID-19 dataset. In datasets with significantly different cell numbers between conditions, cell numbers were downsampled to the same number. Cells were visualized using the ggplot2 package and rasterized for visualization purposes.

### Single-sample GSEA and non-negative matric factorization

To identify signaling pathways responsible for variations observed between cell types and time points, we performed single-sample GSEA (ssGSEA) using the VISION^46^ analysis package. We utilized hallmark, GO and Reactome gene sets from molecular Signature Database (MSigDB)^47^. After calculation of the enrichment of each gene set for each single cell, to cluster similar gene sets into one meta-gene set we measured Pearson’s correlation of different gene sets and clustered highly similar gene sets into one meta-gene set, which we defined as the signature set. Using terms in each signature set we annotated each of them. We performed the same analysis for each of the three lineages studied in this article (HSCs and myeloid cells, B cells and pDCs, and T and NK cell lineages).

To identify the underlying gene program responsible for the generation of infMonos and T_inf_ cells at the 4 h time point of LPS systemic inflammation experiments, we performed non-negative mature factorization (NMF) using the RunNMF function of STutility^48^ package with the default parameters obtaining 40 factors per gene programs. Afterwards, highly similar gene programs were clustered together, generating a meta-gene program. The identified meta-gene program highly enriched in infMonos and T_inf_ cells was visualized using ggplot2. The top genes contributing to each of gene programs are listed in Supplementary Table 5. The same analysis procedure was done to determine gene programs that are highly active in the intermediate and nonclassic monocyte region. Top genes contributing to NCM-enriched and IFN-I gene programs are listed in Supplementary Table 5.

### Analysis of early and late sepsis patient data

For the early sepsis dataset, we downloaded publicly available scRNA-seq data from the Broad Institute Single Cell Portal with the accession no. SCP548 and extracted monocytic and T cell compartments of the data using cell annotations from the corresponding dataset. We performed batch correction for interindividual differences using RunFastMNN function from SeuratWrappers R package and visualized cells using UMAP.

For the late sepsis dataset, we downloaded publicly available scRNA-seq data from the Gene Expression Omnibus (GEO) database with accession no. GSE175453 and extracted the monocytic compartment for the dataset using the cell annotations from the corresponding dataset. We performed batch correction for interdonor differences using the RPCA function from the Seurat package and visualized cells using UMAP. We generated cell density plots for the healthy and late sepsis samples using the embedding_density function of scanpy package.

### Analysis of early COVID-19 or sepsis and late COVID-19 data

For the early mixed COVID-19 or sepsis dataset, we downloaded publicly available scRNA-seq data from the CELLxGENE database (https://cellxgene.cziscience.com/e/ebc2e1ff-c8f9-466a-acf4-9d291afaf8b3.cxg). We extracted monocytic and T cell compartments of the data using cell annotations from the corresponding dataset. We performed batch correction for interdonor differences using the RunFastMNN function from the SeuratWrappers R package.

For the COVID-19 convalescent dataset, we downloaded publicly available scRNA-seq data from the GEO database with accession no. GSE158055. We extracted monocytic compartment from the dataset using the cell annotations from the corresponding dataset. We performed batch correction for interdonor differences using the RunFastMNN function from the SeuratWrappers R package and visualized cells using UMAP.

### Analysis of neutrophil data

For the neutrophil dataset, we downloaded healthy blood and bone marrow data from ArrayExpress under accession no. E-MTAB-11188. We performed batch correction for interindividual differences using the RunFastMNN function from the SeuratWrappers R package and visualized cells using UMAP.

### Reporting summary

Further information on research design is available in the Nature Portfolio Reporting Summary linked to this article.

## Online content

Any methods, additional references, Nature Portfolio reporting summaries, source data, extended data, supplementary information, acknowledgements, peer review information; details of author contributions and competing interests; and statements of data and code availability are available at 10.1038/s41590-025-02136-4.

## Supplementary information

Supplementary Information Supplementary Tables 1 and 4 are merged in this pdf file.

Reporting Summary

Supplementary Table 1 An Excel file containing Supplementary Tables 2, 3 and 5 (each in a separate tab). Supplementary Table 2 Normalized expression of ISGs (obtained from the Reactome database) in monocytes from LPS-challenged participantss. Supplementary Table 3 Response difference (in log_2_(fold-change)) between the first and second LPS challenges. Supplementary Table 5 Top NMF identified genes contributing to iMonos, T_inf_ cells and NCM-enriched and IFN-I gene programs.

## Data Availability

For the early sepsis dataset, we downloaded publicly available scRNA-seq data from the Broad Institute Single Cell Portal with accession no. SCP548. For the late sepsis dataset, we downloaded publicly available scRNA-seq data from the GEO database with accession no. GSE175453. For the early mixed COVID-19 or sepsis dataset, we downloaded publicly available scRNA-seq data from the CELLxGENE database (https://cellxgene.cziscience.com/e/ebc2e1ff-c8f9-466a-acf4-9d291afaf8b3.cxg). For the COVID-19 convalescent dataset, we downloaded publicly available scRNA-seq data from the GEO database with accession no. GSE158055. For the neutrophil dataset, we downloaded healthy blood and bone marrow data from ArrayExpress under accession no. E-MTAB-11188. Bulk RNA-seq and scRNA-seq data for the present study can be downloaded from the GEO database (https://www.ncbi.nlm.nih.gov/geo) with accession no. GSE212093.

## Code availability

Codes used to perform the data analysis related to the present study are available at https://github.com/fkeramati/LPS-systemicinflammation.
