# Supplementary material for: Systemic inflammation impairs myelopoiesis and interferon type I responses in humans
Source: Nat Immunol. 2025 Apr 18;26(5):737–47. doi: 10.1038/s41590-025-02136-4 (PMC12043512; doi:10.1038/s41590-025-02136-4)
Supplement: Supplementary file 1 — Supplementary Tables 1 and 4 are merged in this pdf file. [file 41590_2025_2136_MOESM1_ESM.pdf]

nature immunology

Supplementary information https://doi.org/10.1038/s41590-025-02136-4

Systemic inflammation impairs myelopoiesis and interferon type I responses in humans

In the format provided by the authors and unedited

Supplementary Table 1. Baseline characteristics of healthy male volunteers

	LPS-challenged group (n=7)	Placebo group (n=4)
Age (years)	24 [19-30]	19 [18-28]
Height (cm)	183 [181-187]	186 [174-194]
Weight (kg)	85 [79-91]	83 [72-90]
BMI	26 [24-27]	24 [22-26]
Leukocytes (10^9/L)	6 [5-9]	6 [3-9]

Data in median [range]

Supplementary Table 4. Number of RNA-seq pro led single-cells after quality control

Tissue	Time Point	Donor				
		Donor 1	Donor 2	Donor 3	Donor 4	Total
Bone Marrow	Day 0	6640	7694	4794	-	19128
	4 hours	4224	7562	5469	-	17255
	Day 7	8004	7463	5940	-	21407
PBMC	Day 0	9709	-	9629	7448	26786
	4 hours	5511	-	1425	1762	8698
	Day 7	7366	-	7920	7623	22909
	**Total**	41454	22719	35177	16833	**116183**
