# Supplementary material for: Systemic inflammation impairs myelopoiesis and interferon type I responses in humans
Source: Nat Immunol. 2025 Apr 18;26(5):737–47. doi: 10.1038/s41590-025-02136-4 (PMC12043512; doi:10.1038/s41590-025-02136-4)
Supplement: Supplementary file 2 — Reporting Summary [file 41590_2025_2136_MOESM2_ESM.pdf]

nature portfolio

Corresponding author(s): Hendrik G Stunnenberg, Peter Pickkers, Matthijs Kox

Last updated by author(s): Mar 15, 2025

Reporting Summary

Nature Portfolio wishes to improve the reproducibility of the work that we publish. This form provides structure for consistency and transparency in reporting. For further information on Nature Portfolio policies, see our Editorial Policies and the Editorial Policy Checklist.

Statistics

For all statistical analyses, confirm that the following items are present in the figure legend, table legend, main text, or Methods section.

n/a	Confirmed	
☐	☒	The exact sample size (*n*) for each experimental group/condition, given as a discrete number and unit of measurement
☐	☒	A statement on whether measurements were taken from distinct samples or whether the same sample was measured repeatedly
☐	☒	The statistical test(s) used AND whether they are one- or two-sided<br>*Only common tests should be described solely by name; describe more complex techniques in the Methods section.*
☒	☐	A description of all covariates tested
☐	☒	A description of any assumptions or corrections, such as tests of normality and adjustment for multiple comparisons
☐	☒	A full description of the statistical parameters including central tendency (e.g. means) or other basic estimates (e.g. regression coefficient) AND variation (e.g. standard deviation) or associated estimates of uncertainty (e.g. confidence intervals)
☐	☒	For null hypothesis testing, the test statistic (e.g. *F*, *t*, *r*) with confidence intervals, effect sizes, degrees of freedom and *P* value noted<br>*Give P values as exact values whenever suitable.*
☒	☐	For Bayesian analysis, information on the choice of priors and Markov chain Monte Carlo settings
☒	☐	For hierarchical and complex designs, identification of the appropriate level for tests and full reporting of outcomes
☐	☒	Estimates of effect sizes (e.g. Cohen's *d*, Pearson's *r*), indicating how they were calculated

Our web collection on statistics for biologists contains articles on many of the points above.

Software and code

Policy information about availability of computer code

Data collection	R (4.1.0), Kaluza (2.1)
Data analysis	Following R packages were used to analyze data: Seurat(4.0.4), miloR(2.2.0), msigdbr(7.4.1), clusterProfiler(4.0.5), fgsea(1.18.0), VISION(2.1.0), STutility(0.1.0), scanpy(1.9.1), DESeq2(2.144.0).<br><br>Code used to perform the data analysis related to this study are available at https://github.com/fkeramati/LPS-SI.

For manuscripts utilizing custom algorithms or software that are central to the research but not yet described in published literature, software must be made available to editors and reviewers. We strongly encourage code deposition in a community repository (e.g. GitHub). See the Nature Portfolio guidelines for submitting code & software for further information.

Data

Policy information about availability of data

All manuscripts must include a data availability statement. This statement should provide the following information, where applicable:

- Accession codes, unique identifiers, or web links for publicly available datasets
- A description of any restrictions on data availability
- For clinical datasets or third party data, please ensure that the statement adheres to our policy

For the early sepsis dataset, we downloaded publicly available scRNA-seq data from the Broad Institute Single Cell Portal with the accession number of SCP548. For

the late sepsis dataset, we downloaded publicly available scRNA-seq data from Gene Expression Omnibus (GEO) database with the accession number of GSE175453. For the early mixed COVID-19/sepsis dataset, we downloaded publicly available scRNA-seq data from CELLxGENE database (https://cellxgene.cziscience.com/e/ebc2e1ff-c8f9-466a-acf4-9d291afaf8b3.cxg/). For the COVID-19 convalescent dataset, we downloaded publicly available scRNA-seq data from the GEO database with the accession number of GSE158055. For the neutrophil dataset, we downloaded healthy blood and bone marrow data from ArrayExpress under accession number of E-MTAB-11188.
Bulk RNA-seq and scRNA-seq data for this study can be downloaded from GEO database (https://www.ncbi.nlm.nih.gov/geo/) with the accession number: GSE212093.

Research involving human participants, their data, or biological material

Policy information about studies with human participants or human data. See also policy information about sex, gender (identity/presentation), and sexual orientation and race, ethnicity and racism.

Reporting on sex and gender	Only male subejcts are part of the study. This was based on self-reporting. As human endotoxemia studies are very labor-intensive and expensive, and for ethical reasons (to expose as few volunteers as possible to endotoxemia), nearly all of our LPS studies are restricted to males to increase homogeneity and reduce sample size.
Reporting on race, ethnicity, or other socially relevant groupings	All subjects are white European (Dutch) males.
Population characteristics	Age ranged from 18-30 years. All particpants were healthy, based on medical history, physical examination, laboratory tests and a 12-leads electrocardiogram. All were non-smokers.
Recruitment	Recruitment of healthy volunteers took place by placing advertisements in several faculties and locations on the campus of the Radboud University, Nijmegen and using the Radboud University website. Care was taken into adequately informing the subjects about the burden of participation in the trial. This is a male-only cohort, a limitation already described in the manuscript.
Ethics oversight	This study was approved by the local ethics committee (CMO Arnhem-Nijmegen, The Netherlands, reference no's NL61136.091.17 and 2017-3337). All subjects provided written informed consent.

Note that full information on the approval of the study protocol must also be provided in the manuscript.

Field-specific reporting

Please select the one below that is the best fit for your research. If you are not sure, read the appropriate sections before making your selection.

☒ Life sciences ☐ Behavioural & social sciences ☐ Ecological, evolutionary & environmental sciences

For a reference copy of the document with all sections, see nature.com/documents/nr-reporting-summary-flat.pdf

Life sciences study design

All studies must disclose on these points even when the disclosure is negative.

Sample size	The present study is an explorative study into underlying mechanisms of immune responses in humans and systemic inflammation's long-term effects. As such, a power calculation is not warranted. This was an exploratory study, so no sample size calculation is necessary.
Data exclusions	Data from one donor (in the first section of the study, related to phenotypical characterization of LPS-induced systemic inflammation) was excluded due to outlier results of absolute cell counts.<br>For single-cell data analysis, cells with more than 15% mitochondrial content and number of identified genes below 200 was excluded as low quality cells.<br>Data from two donors in the section related to BST2 flow cytometry analysis (LPS-induced immunosuppression and IFNb reversal) was excluded from the paper, as we did not observe LPS-induced immunosuppression one week after in vivo LPS challenge.
Replication	Information about the replicate numbers are mentioned in the main text and also figure legends. In brief, we used 7 donors for LPS-challenge, 4 donors as placebo and used 3 donors (out of the above mentioned 7 donors) for single-cell RNA-seq and bulk RNA-seq experiments. We recruited 6 donors for ex vivo IFNb reversal experiments. We utilized 8 buffy coats to perform in vitro IFNb reversal experiments. We recruited 3 donors for BST2 flow cytometry analysis of IFNb reversal experiments. We recruited 4 donors for BST2 flow cytometry analysis of monocyte maturation.
Randomization	Subjects were allocated using the sealed envelope method to receive either an intravenous LPS challenge or a placebo challenge with 0.9% NaCl.
Blinding	The study was not blinded since LPS-induced symptoms are very evident for both participants and investigators.

Reporting for specific materials, systems and methods

We require information from authors about some types of materials, experimental systems and methods used in many studies. Here, indicate whether each material, system or method listed is relevant to your study. If you are not sure if a list item applies to your research, read the appropriate section before selecting a response.

Materials & experimental systems

n/a	Involved in the study
☐	☒ Antibodies
☒	☐ Eukaryotic cell lines
☒	☐ Palaeontology and archaeology
☒	☐ Animals and other organisms
☒	☐ Clinical data
☒	☐ Dual use research of concern
☒	☐ Plants

Methods

n/a	Involved in the study
☒	☐ ChIP-seq
☐	☒ Flow cytometry
☒	☐ MRI-based neuroimaging

Antibodies

Antibodies used	Blood was phenotyped with antibodies against CD45-Cy5.5 (A62835, Beckman Coulter), CD14-ECD(B92391, Beckman Coulter), CD16-PE (332779, BD Biosciences), CD64-FITC (B49185, Beckman Coulter), CD11b-PC7 (A54822, Beckman Coulter), HLA-DR-APC (IM3635, Beckman Coulter), DRAQ7 (DR71000, Biostatus), CD192-BV421 (564067, BD Biosciences), CD15-KO (B01176, Beckman Coulter), and dumpgate: CD3-AA750 (A94680, Beckman Coulter) , Mouse Anti Human-Interferon- α/β Receptor Chain 2 Antibody (Merck, MAB1155), CD19-APCA750 (A94681, Beckman Coulter) and CD56-APC A750 (B46024, Beckman Coulter) on a Navios flow cytometer (Beckman Coulter) at the Hematology department of Radboudumc. The surface expression of BST2 using flow cytometry was done using mouse anti human BST2 (CD137/Tetherin) – PE (Beckman Coulter).
Validation	We validated all antibodies based on the respective manufacturer's website referring to the quality of purchased clone of the antibody. All anitbodies used were validated for use in humans according to the manufacturers.

Plants

Seed stocks	Not Relevant
Novel plant genotypes	Not Relevant
Authentication	Not Relevant

Flow Cytometry

Plots

Confirm that:

☒ The axis labels state the marker and fluorochrome used (e.g. CD4-FITC).

☒ The axis scales are clearly visible. Include numbers along axes only for bottom left plot of group (a 'group' is an analysis of identical markers).

☒ All plots are contour plots with outliers or pseudocolor plots.

☒ A numerical value for number of cells or percentage (with statistics) is provided.

Methodology

Sample preparation	EDTA-anticoagulated blood was stained with antibodies for 15 min at 4 degrees Celsius. Subsequently, erythrocytes were lysed (NH4CL: 180 mL + 20 mL lysis stock dilution [BD Pharm-Lyse, BectonDickinson]) and cells were washed three times in PBS before analysis on a Navios flow cytometer (Beckman Coulter).
Instrument	Navios flow cytometer (Beckman Coulter)
Software	Kaluza V2.1
Cell population abundance	For relative abundance of monocyte subtypes in peripheral blood, after gating and excluding non-monocyte cells, the relative abundance of each monocyte subtype (classical, intermediate and non-classical) was determined based on the abundance of each respective population relative to all monocytes. Absolute quantities of the subsets were subsequently calculated by

multiplying their relative abundance by the total monocytes count in peripheral blood determined using a Sysmex XE-5000 instument (Sysmex).

Gating strategy

Cells -> Singlets -> DRAQ7- cells -> CD64+ -> Determine monocyte subtypes based on the expression of CD14 and CD16. Full gating strategy to determine monocyte sub-populations is depicted in Extended Figure 8a.

☒ Tick this box to confirm that a figure exemplifying the gating strategy is provided in the Supplementary Information.
